# B7-H3 upregulation in ischemic stroke: friend or foe?

**DOI:** 10.1016/j.expneurol.2026.115675

**Published:** 2026-01-29

**Authors:** Siva Reddy Challa, Isidra M. Baker, Casimir A. Fornal, Sahil Reddy Mada, Nabeeha Khan, Samantha N. Jackson, Erick Saldes, Jeffrey D. Klopfenstein, Swapna Asuthkar, Krishna Kumar Veeravalli

**Affiliations:** a Department of Cancer Biology and Pharmacology, University of Illinois College of Medicine at Peoria, Peoria, IL, USA; b Department of Neurosurgery, University of Illinois College of Medicine at Peoria, Peoria, IL, USA; c Department of Pediatrics, University of Illinois College of Medicine at Peoria, Peoria, IL, USA; d Department of Neurology, University of Illinois College of Medicine at Peoria, Peoria, IL, USA

**Keywords:** B7-H3, Ischemia, Reperfusion, Stroke, Inflammation

## Abstract

B7-H3 (CD276) is an immune checkpoint co-signaling molecule expressed on immune and non-immune cells. It is best known for suppressing T-cell responses but can also promote inflammation depending on the microenvironment. In neuroinflammatory models such as experimental autoimmune encephalomyelitis, B7-H3 expression increases concomitantly with the inflammatory response, and its inhibition is associated with reduced disease progression. Although its role in ischemic stroke remains unclear, we hypothesized that cerebral ischemia/reperfusion (I/R) would upregulate B7-H3 expression in the ischemic brain and that increased B7-H3 expression would positively correlate with pro-inflammatory cytokine expression. Young and aged male and female rodents, including normotensive and spontaneously hypertensive rats to model comorbid hypertension, underwent transient middle cerebral artery occlusion (MCAO) followed by reperfusion. Brain tissue was collected on post-MCAO days 1, 3, 5, or 7. B7-H3 mRNA was analyzed by real-time PCR, whereas protein expression was assessed by Western blotting and immunohistochemistry at selected time points. B7-H3 expression was significantly upregulated in the ischemic brain across sexes, age groups, and species. The extent of B7-H3 degradation was influenced by species, sex, age, and time after cerebral I/R. Upregulation of B7-H3 was observed at both the mRNA and protein levels and was localized primarily to the somatosensory cortex and caudate putamen in the ipsilateral (ischemic) hemisphere, the main regions affected in this MCAO model. Elevated B7-H3 expression in the ischemic brain positively correlated with the pro-inflammatory mediator TNFα. In rats, the temporal profile of B7-H3 expression paralleled the early inflammatory phase associated with secondary tissue damage after ischemic stroke. These findings identify B7-H3 as an ischemia-induced immune checkpoint molecule in the brain that may modulate post-stroke immune responses and support further investigation into its beneficial versus detrimental roles in neuroinflammation and its potential as a therapeutic target following cerebral I/R.

## 1. Introduction

Stroke is currently the fifth leading cause of death in the United States and the second leading cause of death globally (Furie, 2020). Ischemic stroke is the most prevalent type, accounting for approximately 87% of all strokes (Virani et al., 2020). The currently available recanalization treatment options for a subset of patients with acute ischemic stroke are (1) thrombolytic drugs that dissolve intravascular clots, namely tissue-type plasminogen activator and tenecteplase, and (2) endovascular thrombectomy, a minimally invasive procedure for clot removal. These interventions reperfuse the previously ischemic tissue; however, reperfusion can also precipitate secondary brain damage, termed reperfusion injury. Therapies that mitigate progressive secondary brain injury after reperfusion remain an unmet clinical need.

Advanced age and vascular comorbidities such as hypertension are major risk factors that increase both the incidence and severity of ischemic stroke (Fisher et al., 2007; Howells et al., 2010; O’Donnell et al., 2010; Mozaffarian et al., 2015). Hypertensive animals and aged rodents therefore provide clinically relevant models for investigating mechanisms that differentially modulate stroke injury and recovery in high-risk populations.

Harmful neuroinflammation triggered by aberrant immune activation following cerebral ischemia/reperfusion (I/R) critically exacerbates brain damage and impedes functional recovery (Jayaraj et al., 2019). GEO database analyses indicate that several immune-related genes, including CD276 (B7-H3), are highly expressed in stroke patient samples (Tao et al., 2022). B7-H3 is an immune checkpoint co-signaling molecule expressed on both immune and non-immune cells that plays a multifaceted role at the interface of innate and adaptive immunity. B7-H3 can function as both a T-cell costimulatory and coinhibitory molecule. As a costimulatory molecule, B7-H3 enhances T-cell proliferation and IFNγ production and augments pro-inflammatory cytokine release from monocytes/macrophages (Chapoval et al., 2001; Zhang et al., 2010). Consistent with this pro-inflammatory role, upregulation of B7-H3 has been reported to enhance inflammatory signaling, increase cytokine production, and promote blood-brain barrier disruption (Chen et al., 2017), while B7-H3 knockout mice exhibit reduced inflammation in models of collagen-induced arthritis and experimental autoimmune encephalomyelitis (Luo et al., 2015). In contrast, B7-H3 functions predominantly as a coinhibitory molecule in the context of cancer (Castellanos et al., 2017; Kontos et al., 2021; Getu et al., 2023). Together, these reports illustrate the functional duality of B7-H3, which may exert costimulatory or coinhibitory effects depending on the context and the microenvironment, in part through differential modulation of distinct T-cell subsets. For example, B7-H3 can enhance Th1/Th17 responses while suppressing Th2 responses, depending on the predominant T-cell subsets within the microenvironment (Luo et al., 2015). Despite the known role of B7-H3 in immune regulation and its detection in stroke patient samples, no studies have systematically characterized B7-H3 expression in preclinical stroke models across rodent species, sex, age, and comorbidities, and neither its temporal expression profile nor its regional localization has been determined.

This study aims to characterize B7-H3 expression in rodent stroke models across species, sex, age, and time points following cerebral I/R. By defining the temporal expression profiles and regional localization of B7-H3 upregulation in these models, we sought to identify suitable preclinical rodent models and to delineate an optimal treatment window for future evaluation of therapies targeting B7-H3. We also examined B7-H3 expression in normotensive Wistar-Kyoto (WKY) rats and spontaneously hypertensive rats (SHRs) to assess whether B7-H3 upregulation is accentuated in hypertensive animals that develop more severe neurological deficits after middle cerebral artery occlusion (MCAO). In addition, we analyzed the relationship between B7-H3 expression and the expression levels of three pro-inflammatory cytokines (IL-1β, IL-6, and TNFα) in the ischemic brain.

## 2. Material and methods

### 2.1. Ethics and compliance statements

All animal procedures were conducted in accordance with a protocol (Protocol #1284641) approved by the Institutional Animal Care and Use Committee at the University of Illinois College of Medicine Peoria (UICOMP). All experiments adhered to the scientific, humane, and ethical principles of UICOMP and to the guidelines outlined in the *Guide for the Care and Use of Laboratory Animals* (NIH Publication No. 86–23, revised; U.S. Department of Health and Human Services). Animal experiments were designed, conducted, and reported in accordance with the Animal Research: Reporting of In Vivo Experiments (ARRIVE) guidelines (Percie du Sert et al., 2020).

### 2.2. Animals and experimental groups

Sprague-Dawley (SD) rats were obtained from Envigo (Indianapolis, IN, USA). Wistar-Kyoto (WKY) rats and spontaneously hypertensive rats (SHRs) were obtained from Charles River (Wilmington, MA, USA). C57BL/6J mice were obtained from The Jackson Laboratory (Bar Harbor, ME, USA). Rats and mice were housed in the UICOMP Laboratory Animal Care Facility and maintained under controlled temperature and humidity on a 12-h light/dark cycle with ad libitum access to food and water. A total of 59 young (3-month-old) male SD rats, 14 young (3-month-old) male WKY rats, 24 young (3-month-old) male SHRs, and 59 C57BL/6 J mice (both sexes combined; young [3-month-old] and aged [17–19-month-old]) were used in this study and were randomly assigned to either Control or Stroke groups, as summarized in Supplementary Table 1.

### 2.3. Stroke induction in rats

Stroke induction in rats was performed by right transient middle cerebral artery occlusion (MCAO) using the intraluminal monofilament suture technique. Rats were deeply anesthetized with 3.0–3.5% isoflurane delivered via a VetFlo isoflurane anesthesia system (Kent Scientific Corporation, Torrington, CT, USA) and maintained at 2.5–3.0% isoflurane throughout surgery. Under anesthesia, rats were placed on a SurgiSuite multifunctional surgical platform with integrated far-infrared warming (Kent Scientific Corporation, Torrington, CT, USA) to maintain normothermia and prevent hypothermia during surgery. An aseptic technique was followed throughout the procedure. A ventral midline neck incision (~25 mm) was made, and the right common carotid artery (CCA), internal carotid artery (ICA), and external carotid artery (ECA) were surgically exposed. The ECA was permanently ligated rostrally with a single 5–0 silk suture, and a second loose ligature was placed around the ECA near the CCA bifurcation. Microaneurysm clips were applied to the CCA and ICA. The ECA was then transected between the two ligatures. An appropriately sized silicone rubber-coated monofilament suture (Doccol Corporation, Sharon, MA, USA) was introduced through the stump of the severed ECA and advanced into the ICA. The microaneurysm clip was removed from the ICA, and the monofilament was gently advanced 18–19 mm to a premarked distance corresponding to the origin of the middle cerebral artery (MCA). The loose ligature near the bifurcation was then tightened around the ECA containing the monofilament. The microaneurysm clip on the CCA was removed, and the neck incision was closed with surgical wound clips. To restore blood flow, reperfusion was initiated 2 hours after MCAO in SD rats and 1 hour after MCAO in WKY rats and SHRs. The neck incision was reopened by removing the wound clips, and a microaneurysm clip was reapplied to the CCA. The ECA ligature was loosened, the monofilament suture was carefully withdrawn, and the ligature was retied to achieve hemostasis. The microaneurysm clip on the CCA was then removed, and the neck incision was closed with 3–0 nylon sutures. Rats in the control group underwent surgical procedures identical to the MCAO surgery, except that the monofilament was not inserted. Throughout this manuscript, “control” refers to these sham-operated animals.

Pre- and post-surgical care was provided in accordance with the IMPROVE guidelines (*Ischemia Models: Procedural Refinements of* In Vivo *Experiments*) (Percie du Sert et al., 2017). Post-surgical care for rats included administration of the analgesic carprofen (5 mg/kg, s.c., once daily for 2 days following surgery), and subcutaneous sterile saline (approximately 2 mL on the day of surgery and for 2 days thereafter) to maintain hydration. A topical triple-antibiotic ointment containing bacitracin, neomycin, and polymyxin B was applied prophylactically to the neck incision to prevent infection. In addition, a few dry and moistened food pellets were placed on the cage floor to facilitate access to food during recovery.

### 2.4. Modified neurological severity score (mNSS) assessment in rats

The mNSS is a composite index of motor, sensory, reflex and balance function (Chen et al., 2001). Neurological deficits were assessed using the mNSS in rats at 2–4 h and again on post-MCAO day 1 to determine stroke severity. Scores range from 0 (no deficit) to 18 (maximal neurological impairment).

### 2.5. Stroke induction in mice

Stroke induction in mice was performed using the same right transient MCAO monofilament technique as in rats, with minor modifications. Mice were deeply anesthetized with 3% isoflurane delivered via a VetFlo isoflurane anesthesia system (Kent Scientific Corporation, Torrington, CT, USA) and maintained at 2% isoflurane throughout surgery. Under anesthesia, mice were placed on a SurgiSuite multifunctional surgical platform with integrated far-infrared warming (Kent Scientific Corporation, Torrington, CT, USA) to maintain normothermia and prevent hypothermia during surgery. An aseptic technique was followed throughout the procedure. A ventral midline neck incision (10–15 mm) was made, and the right CCA, ICA, and ECA were surgically exposed. The ECA was permanently ligated rostrally with a single 6–0 silk suture, and a second loose ligature was placed around the ECA near the CCA bifurcation. Blood flow in the CCA and ICA was temporarily occluded by lifting each vessel with 5–0 silk sutures. The ECA was then transected between the two ligatures. An appropriately sized silicone rubber-coated monofilament suture (Doccol Corporation, Sharon, MA, USA) was introduced through the stump of the severed ECA and advanced into the ICA while tension on the ICA ligature was slowly released. The monofilament was gently advanced approximately 8–9 mm to a premarked distance corresponding to the origin of MCA. The loose ligature was then tightened around the ECA stump containing the monofilament. Tension on the CCA was relieved by removing the silk suture, and the skin incision was temporarily closed with surgical wound clips. Following completion of the initial surgery, mice were allowed to recover from anesthesia in a surgical recovery cage equipped with a water-perfused heating pad beneath one half of the cage to allow behavioral thermo-regulation. Mice were re-anesthetized with isoflurane shortly before the designated reperfusion time (1 hour after MCAO). The neck incision was reopened by removing the wound clips, and the CCA was again lifted with a 5–0 silk suture to temporarily block blood flow. The ECA ligature was loosened, the monofilament suture was carefully withdrawn to initiate reperfusion, and the ligature was retightened to achieve hemostasis. Tension on the CCA was then relieved, and the neck incision was closed with 5–0 nylon sutures. Mice in the control group underwent surgical procedures identical to the MCAO surgery, except that the monofilament was not inserted. Thus, “control” refers to sham-operated animals.

Pre- and post-surgical care was provided in accordance with the IMPROVE guidelines (*Ischemia Models: Procedural Refinements of* In Vivo *Experiments*) (Percie du Sert et al., 2017). Post-surgical care for mice included administration of the analgesic carprofen (5 mg/kg, s.c., once daily for 2 days following surgery), and subcutaneous sterile saline (approximately 0.5 mL on the day of surgery and for 2 days thereafter) to maintain hydration. A topical triple-antibiotic ointment containing bacitracin, neomycin, and polymyxin B was applied prophylactically to the neck incision to prevent infection. In addition, a few dry and moistened food pellets were placed on the cage floor to facilitate access to food during recovery.

### 2.6. Neurological deficit score (NDS) assessment in mice

Neurological deficit score (NDS) assessments were conducted in mice at 2–4 h and again on post-MCAO day 1 to determine stroke severity. Neurological function was graded on a 0–3 scale as follows: 0, no observable deficits; 1, forelimb flexion; 2, forelimb flexion and decreased resistance to lateral push; 3, forelimb flexion, decreased resistance to lateral push, and circling.

### 2.7. Exclusion criteria

Animals that died or were euthanized during the study period, as well as those exhibiting post-mortem bleeding in the region of the MCA, were excluded from the study. In addition, mice with NDS scores <2 at either 2–4 h or on day 1 post-MCAO were excluded. Finally, statistical outliers were excluded using the ROUT test (Q = 1%).

### 2.8. Tissue collection and processing

Brain tissue was collected from rats and mice from both control and stroke groups. Brains were harvested from rats on post-MCAO days 1, 3, 5, or 7, and from mice on post-MCAO days 1 and 3. Under deep anesthesia, animals were transcardially perfused, and brains were collected for molecular and histological analyses as described below.

For real-time PCR and Western blot analyses in rats, animals were perfused intracardially with ice-cold 1× phosphate-buffered saline (PBS) to remove intravascular blood. Brains were rapidly removed, and the ipsilateral (ischemic) hemisphere was separated from the contralateral hemisphere and stored at −80 °C until processing for RNA isolation and protein extraction. For immunohistochemical analysis in rats, animals were perfused intracardially with PBS followed by 10% neutral buffered formalin. Brains were post-fixed in the same fixative for 24 h at 4 °C and then cryoprotected in 30% (*w*/*v*) sucrose in PBS until they sank. Cryoprotected brains were embedded in optimal cutting temperature (OCT) compound (Tissue-Tek OCT, Catalog #4583; Sakura Finetek USA, Torrance, CA, USA), frozen, and stored at −80 °C until sectioning. Coronal brain sections (40 μm thick) were cut on a cryostat (Leica CM1950; Leica Biosystems), and sections containing the caudate putamen at the level of the anterior commissure were collected into 24-well polystyrene plates containing PBS with 0.02% sodium azide (as a preservative) and stored at 4 °C for DAB-based immunohistochemistry.

For Western blot analysis in mice, animals were perfused intracardially with ice-cold PBS as described above. Brains were removed, the ipsilateral (ischemic) hemisphere was dissected, and tissue was stored at −80 °C until protein extraction.

### 2.9. RNA isolation and cDNA synthesis

Total RNA was extracted from the entire ipsilateral (ischemic) cerebral hemispheres of rats from both control and stroke groups using TRIzol Reagent (Invitrogen, Carlsbad, CA, USA). One microgram of total RNA from each sample was reverse-transcribed into cDNA using iScript cDNA Synthesis Kit (Bio-Rad Laboratories, Hercules, CA, USA), and the resulting cDNA was diluted 1:10 in nuclease-free water and stored at −20 °C for subsequent analysis.

### 2.10. Real-time PCR analysis

For each diluted (1:10) cDNA sample, reactions were assembled using the iTaq Universal SYBR Green Supermix (Bio-Rad Laboratories, Hercules, CA, USA) according to the manufacturer’s instructions. Forward and reverse primers for the target genes (Integrated DNA Technologies, Coralville, IA, USA) were diluted 1:10 in nuclease-free water. Primer sequences for the target genes are listed in Supplementary Table 2. PCR reactions were performed in triplicates using the following thermal cycling conditions: initial denaturation at 95 °C for 5 min; 40 cycles of 95 °C for 30 sec, 60 °C for 30 sec, and 72 °C for 30 sec; and a final extension at 72 °C for 5 min. Reactions were run on an iCycler IQ Multi-Color Real-Time PCR detection system (Bio-Rad Laboratories, Hercules, CA, USA). Data were collected using iCycler IQ software (Bio-Rad Laboratories, Hercules, CA, USA) and expressed as threshold cycle (Ct) values, defined as the number of cycles at which the fluorescent intensity of the SYBR Green dye exceeded background fluorescence. For each sample, the mean Ct value from triplicate reactions (after removal of any outlier), was used for analysis. *18S* rRNA served as the internal reference gene. Relative target gene expression was normalized to *18S* rRNA, and fold change (stroke relative to control) was calculated as 2^ (ΔCt control)/2^(ΔCt test), where ΔCt = Ct (target) - Ct (*18S* rRNA).

### 2.11. Western blot analysis

Western blot analysis was performed using brain tissue lysates prepared from the entire ipsilateral (ischemic) cerebral hemispheres of rats and mice in control and stroke groups collected on post-MCAO days 1 or 3. Tissue was homogenized in RIPA Lysis and Extraction Buffer (Thermo Fisher Scientific, Waltham, MA, USA), and protein concentrations were determined using the Pierce Bicinchoninic Acid (BCA) Protein Assay Kit (Thermo Fisher Scientific, Waltham, MA, USA). Equal amounts of protein from each sample were loaded into individual wells of a SurePAGE Bis-Tris precast gel (GenScript, Piscataway, NJ, USA) alongside a BLUEstain Protein Ladder (Gold Biotechnology, Olivette, MO, USA) and subjected to sodium dodecyl sulfate-polyacrylamide gel electrophoresis (SDS-PAGE). Separated proteins were then transferred onto a 0.22-μm nitrocellulose membrane (Fisher Scientific, Hampton, NH, USA). Membranes were blocked with 5% non-fat dry milk in PBST (1× PBS containing 0.1% Tween-20) and probed with a mouse monoclonal anti-B7-H3 antibody (Catalog #sc-376769; Santa Cruz Biotechnology, Dallas, TX, USA) followed by an HRP-conjugated goat anti-mouse IgG secondary antibody. Each membrane was then reprobed with a mouse monoclonal anti-GAPDH antibody (Catalog #sc-32233; Santa Cruz Biotechnology, Dallas, TX, USA) followed by incubation with the same HRP-conjugated goat anti-mouse IgG secondary antibody. Immunore-active bands were visualized using an enhanced chemiluminescence (ECL) Western blot detection reagent (Bio-Rad Laboratories, Hercules, CA, USA) on a KwikQuant Digital Western Blot Detection System (Kindle Biosciences, Greenwich, CT, USA). Band intensities were quantified using NIH ImageJ software (version 1.54p) and normalized to the loading control, GAPDH.

### 2.12. Immunohistochemistry

For each rat, a single coronal brain section through the caudate putamen at the level of the anterior commissure was processed for immunohistochemistry. Coronal sections were mounted onto adhesive microscope slides (Catalog #1354W; Diamond White Glass, Globe Scientific, Mahwah, NJ, USA) from 50 mM phosphate buffer (pH 7.4). Slides were dried overnight at 37 °C and then left at room temperature for an additional day prior to processing. Slide-mounted sections were subjected to heat-induced epitope retrieval in 10 mM citric acid (pH 6.0) at 90 °C for 30 min using a Precision water bath (Model 282; Winchester, VA, USA). Following rinses with 1× PBS, sections were incubated in 0.3% hydrogen peroxide in PBS for 30 min to quench endogenous peroxidase activity. Sections were then permeabilized with 0.1% Tween-20 in PBS for 30 min and blocked for 60 min with 5% normal goat serum containing 0.05% Tween-20 in PBS. After blocking, sections were incubated overnight at 4 °C with a mouse monoclonal anti-B7-H3 antibody (Catalog #sc-376769; Santa Cruz Biotechnology, Dallas, TX, USA) at a 1:100 dilution in 3% normal goat serum containing 0.05% Tween-20 in PBS. The following day, sections were rinsed with PBS and incubated for 1 h with an HRP polymer-conjugated goat anti-mouse IgG secondary antibody (Catalog #VC001; R&D Systems, Minneapolis, MN, USA). After additional rinses with PBS, sections were reacted with 3,3′-diaminobenzidine (DAB) using the SigmaFAST tablet set (Catalog #D4418; Sigma-Aldrich, St. Louis, MO, USA) containing urea hydrogen peroxide as the oxidant for HRP. Once optimal DAB staining intensity was achieved, the reaction was stopped by rinsing the sections in deionized water. Slides were dehydrated through a graded ethanol series (50%, 70%, 95%, and 100%), cleared in two changes of xylene, and coverslipped with DPX mounting medium (Catalog #360294H; VWR, USA).

After the slides were air-dried, they were cleaned and scanned at 10,000 dpi using a PrimeHisto XE slide scanner (Pacific Image Electronics, Taiwan). Digital images were analyzed using NIH ImageJ software (version 1.54p). Each image was imported and converted to 8-bit format. The integrated density of B7-H3 staining (stained area × mean grayscale value) was quantified separately in the contralateral (non-ischemic) and ipsilateral (ischemic) hemispheres. A fixed threshold range (minimum and maximum grayscale values) corresponding to the B7-H3-positive signal was established and applied uniformly to all sections to ensure consistency and comparability between treatment groups. Because B7-H3-positive staining appeared as darker pixels with lower grayscale values, the measured grayscale values within the thresholded region were numerically inverted so that higher values corresponded to stronger staining. These inverted values were used for data analysis.

### 2.13. Statistical analysis

Statistical analysis of the data was performed using GraphPad Prism 10.4.2 for Windows (GraphPad Software, San Diego, CA, USA). Outliers in the data were identified using the ROUT test (Q = 1%) and excluded from the analysis. Quantitative data from each experiment were tested for normality (Shapiro-Wilk test) and equality of variances (F-test and Bartlett’s test). Based on the number of groups in each experiment and the results of the normality and variance tests, appropriate statistical tests were applied, including Mann-Whitney test when normality was not met, a two-tailed unpaired *t*-test with Welch’s correction when variances were unequal, one-way ANOVA followed by Dunnett’s multiple comparisons test, and two-way ANOVA followed by Sidak’s multiple comparisons test. Pearson’s correlation test (Pearson’s *r*) was used to assess relationships between variables. Differences between groups were considered statistically significant at *p* < 0.05. All data are presented as mean ± SEM.

## 3. Results

### 3.1. B7-H3 expression increases in the brain after ischemic stroke in young rats

**Fig. 1.**
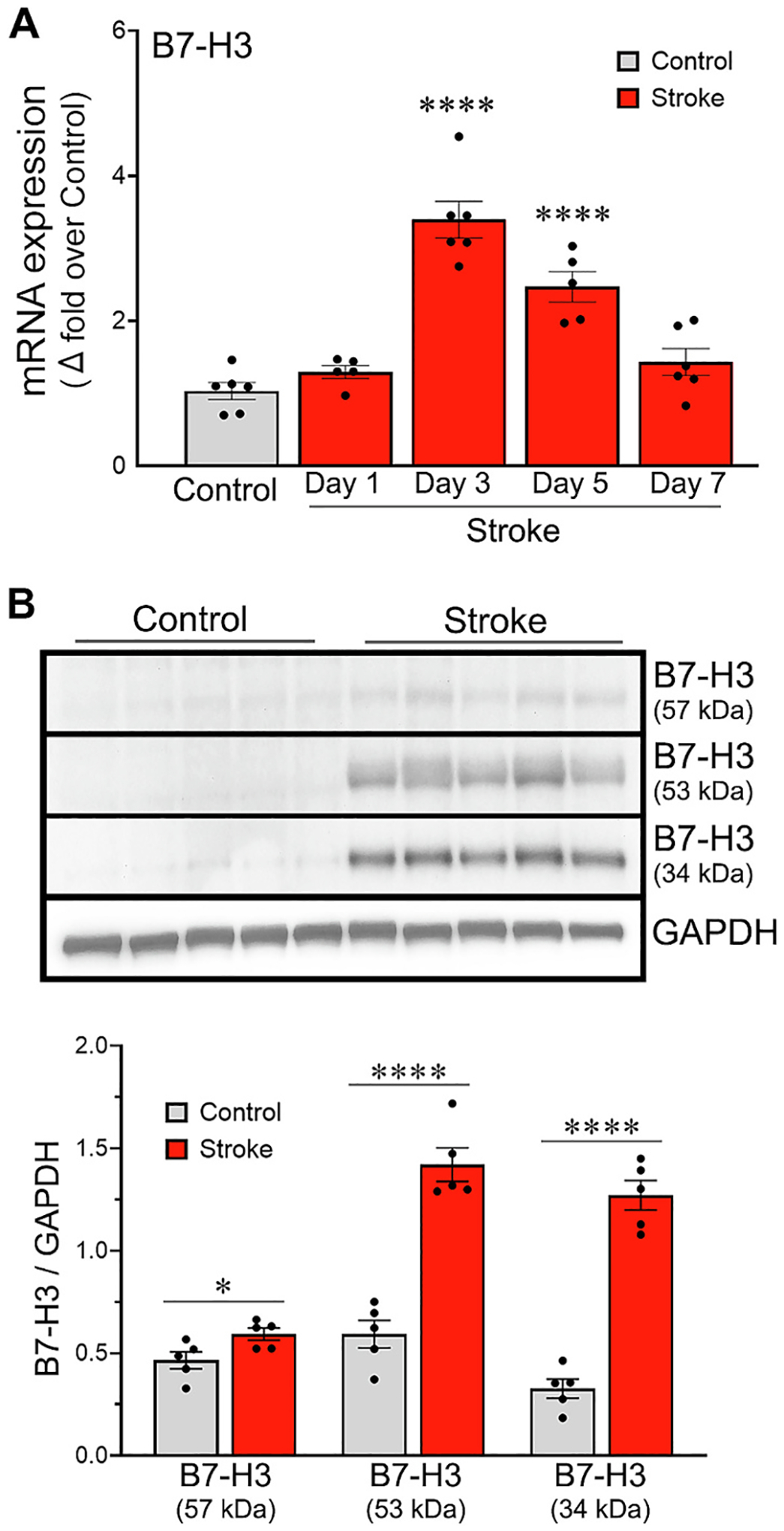
B7-H3 expression increases in the brain after ischemic stroke in young rats (2-h MCAO followed by reperfusion). (A) Column scatter plot shows B7-H3 mRNA expression in the ipsilateral (ischemic) hemisphere on post-MCAO days 1, 3, 5, and 7. Error bars indicate SEM; *n* = 5–6/group. ****p < 0.0001 versus control. (B) Immunoblots show increased B7-H3-immunoreactve bands (57, 53, and 34 kDa) in the ipsilateral (ischemic) hemisphere on post-MCAO day 3. Column scatter plot shows the quantified expression of the 57-, 53-, and 34-kDa bands normalized to GAPDH. Error bars indicate SEM; n = 5/group. *p < 0.05, ****p < 0.0001.

Cerebral ischemia/reperfusion (I/R) induced by transient middle cerebral artery occlusion (MCAO) in young male Sprague-Dawley (SD) rats resulted in a time-dependent increase in B7-H3 mRNA expression in the ipsilateral (ischemic) hemisphere, with mean levels higher on post-MCAO days 1, 3, 5, and 7 compared with the control group (Fig. 1A). Across these post-MCAO days B7-H3 upregulation was modest on day 1, peaked on day 3, declined but remained elevated on day 5, and further declined toward baseline by day 7. However, one-way ANOVA followed by Dunnett’s multiple comparisons test showed that these increases were statistically significant only on days 3 and 5 (both *p* < 0.0001), whereas changes on days 1 and 7 did not reach significance. Western blot analysis of ipsilateral (ischemic) hemisphere samples collected on post-MCAO day 3 showed increased B7-H3-immunoreactive bands (57, 53, and 34 kDa) in the stroke group relative to controls (Fig. 1B). A two-tailed unpaired *t*-test confirmed that the 57-kDa band was significantly increased (*p* = 0.0363) and that the 53-kDa and 34-kDa bands were markedly upregulated (both *p* < 0.0001) in the stroke group compared with the control group.

### 3.2. B7-H3 expression increases in the ischemic brain of both sexes of young mice

**Fig. 2.**
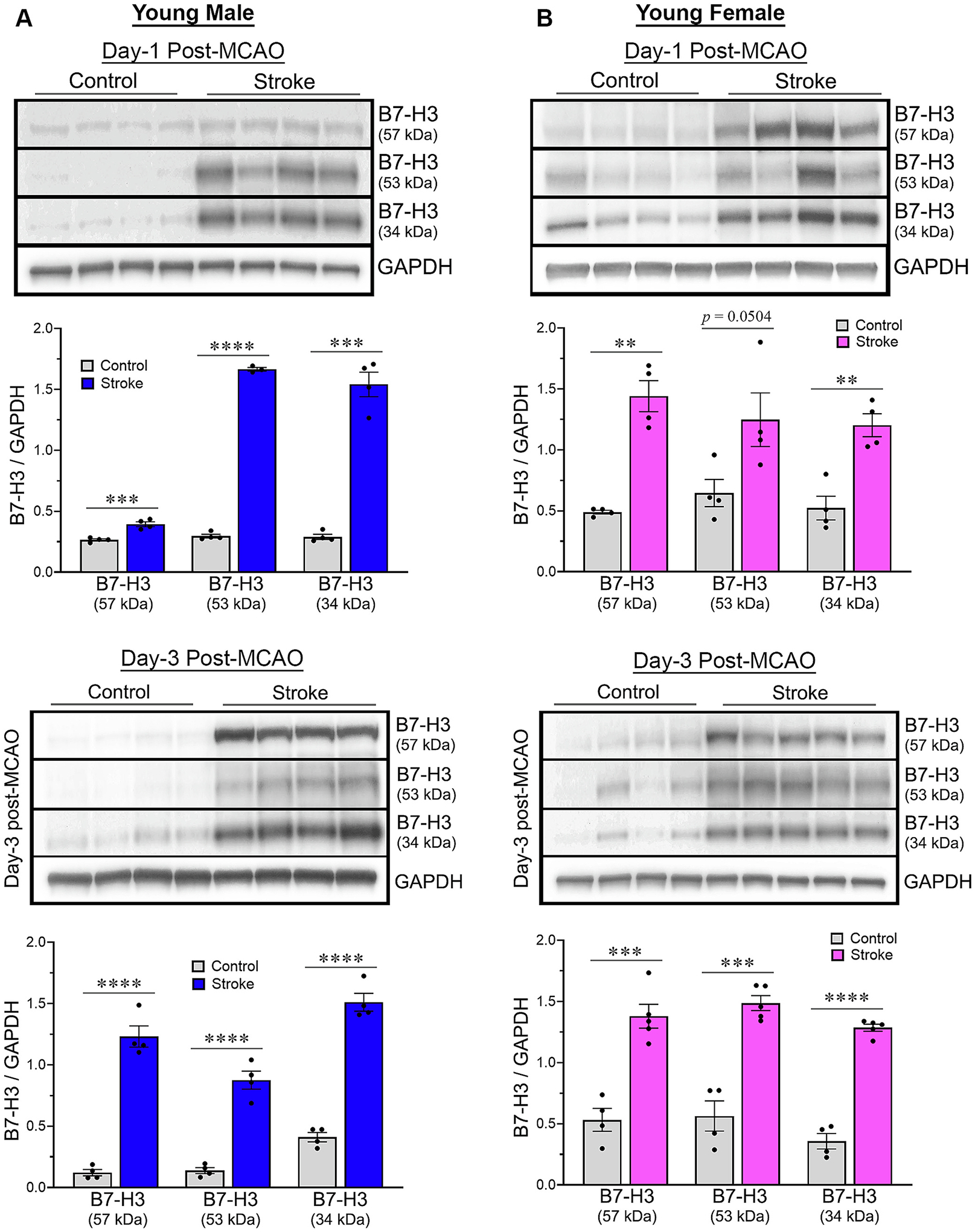
B7-H3 protein expression increases in the brain after ischemic stroke in young male and female mice (1-h MCAO followed by reperfusion). Immunoblots show B7-H3-immunoreactive bands (57, 53, and 34 kDa) in the ipsilateral (ischemic) hemispheres of young male (A) and female (B) mice compared with age-matched controls on post-MCAO days 1 and 3. Column scatter plots show the quantified expression of the 57-, 53-, and 34-kDa bands normalized to GAPDH. Error bars indicate SEM; *n* = 4–5/group. **p < 0.01, ***p < 0.001, ****p < 0.0001.

To determine whether the ischemia-induced B7-H3 upregulation observed in rats extends to a second rodent species, we examined B7-H3 protein expression in the ipsilateral (ischemic) hemispheres of young male and female mice subjected to 1-h MCAO followed by reperfusion. Western blot analysis of brain samples collected on post-MCAO days 1 and 3 showed increased B7-H3 expression in the stroke groups compared with their respective control groups (Fig. 2). In young males, two-tailed unpaired *t*-tests (with Welch’s correction applied when variances were unequal) revealed significantly higher B7-H3 protein levels in the stroke group than in controls on post-MCAO day 1 for the 57-kDa band (*p* = 0.0009), 53-kDa band (*p* < 0.0001), and the 34-kDa band (*p* = 0.0008). On post-MCAO day 3, all three bands were significantly increased (*p* < 0.0001 for each) (Fig. 2A). Similarly, in young females, two-tailed unpaired t-tests (with Welch’s correction applied when variances were unequal) showed significantly higher B7-H3 protein expression in the stroke groups than in controls on post-MCAO day 1 for the 57-kDa band (*p* = 0.0046) and the 34-kDa band (*p* = 0.0024), whereas the 53-kDa band did not reach statistical significance (*p* = 0.0504). On post-MCAO day 3, all three bands were significantly increased (57-kDa, *p* = 0.0005; 53-kDa, *p* = 0.0002; 34-kDa, *p* < 0.0001) (Fig. 2B).

### 3.3. B7-H3 expression increases in hypertensive and aged rodent models of cerebral ischemia/reperfusion

**Fig. 3.**
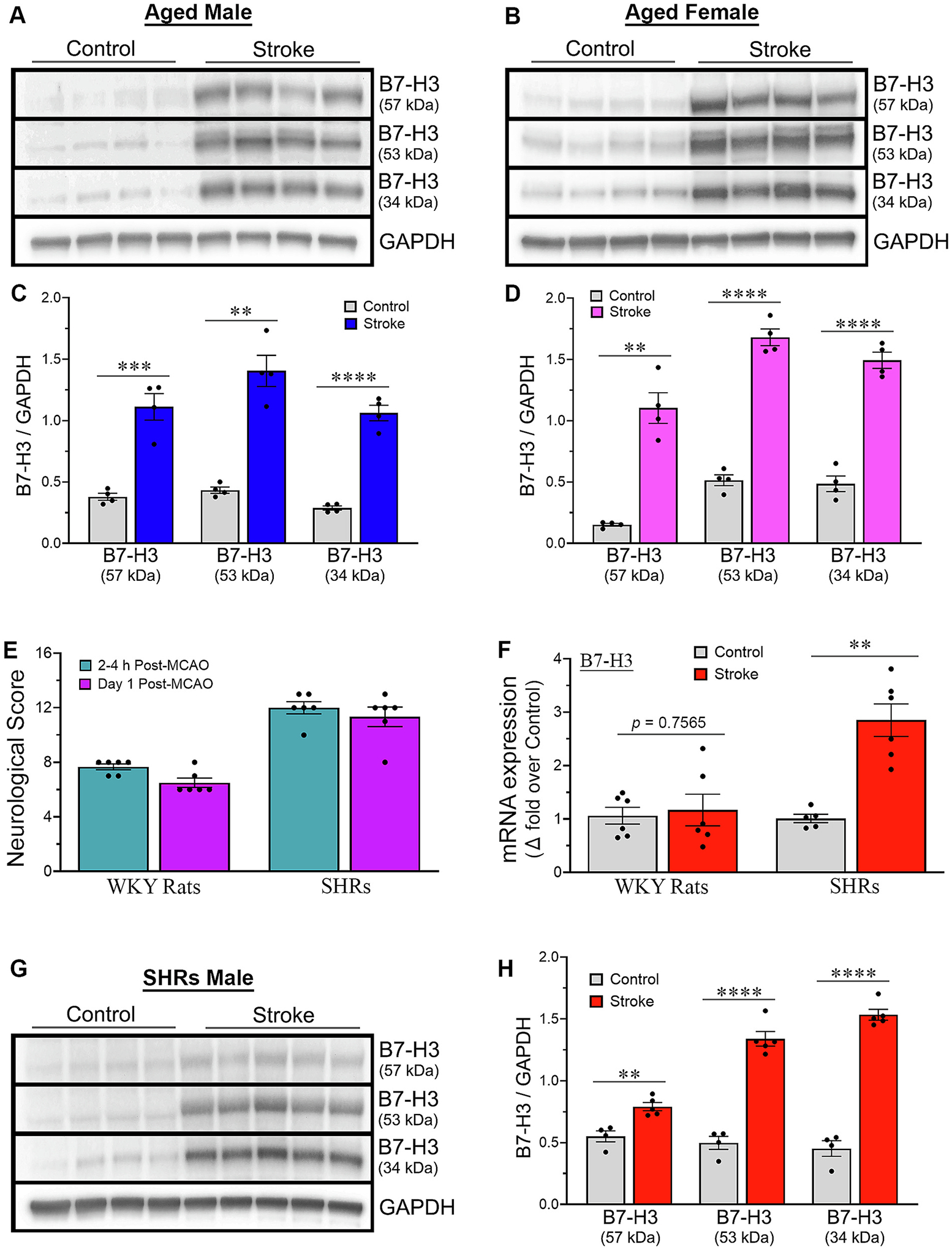
B7-H3 expression increases in the brain after ischemic stroke in clinically relevant rodent models (1-h MCAO followed by reperfusion). (A, B) Immunoblots show increased B7-H3-imunoreactive bands (57, 53, and 34 kDa) in the ipsilateral (ischemic) hemispheres of aged male (A) and female (B) mice compared with age-matched controls on post-MCAO day 1. (C, D) Column scatter plots show the quantified expression of the 57-, 53-, and 34-kDa bands in aged male (C) and female (D) mice normalized to GAPDH. Error bars indicate SEM; *n* = 4/group. **p < 0.01, ***p < 0.001, ****p < 0.0001. (E) Column scatter plot shows modified neurological severity scores (mNSS) in male WKY rats and SHRs on post-MCAO day 1. Error bars indicate SEM; *n* = 6/group. (F) Column scatter plot shows B7-H3 mRNA expression in male WKY rats and SHRs on post-MCAO day 3. Error bars indicate SEM; *n* = 5–6/group. **p < 0.01. (G) Immunoblots show increased B7-H3-immunoreactive bands (57, 53, and 34 kDa) in the ipsilateral (ischemic) hemispheres of male SHRs compared with controls on post-MCAO day 3. (H) Column scatter plot shows the quantified expression of the 57-, 53-, and 34-kDa bands in SHRs normalized to GAPDH. Error bars indicate SEM; n = 4–5/group. **p < 0.01, ****p < 0.0001.

Because advanced age, hypertension, and sex influence stroke risk and severity, we next examined B7-H3 protein expression in more clinically relevant models: spontaneously hypertensive rats (SHRs) and aged male and female mice subjected to 1-h MCAO followed by reperfusion. Western blot analysis of ipsilateral (ischemic) hemisphere samples collected on post-MCAO day 1 from aged male and female mice showed increased B7-H3 expression in the stroke groups compared with their respective control groups (Fig. 3A, B). In aged males, two-tailed unpaired *t*-tests (with Welch’s correction applied when variances were unequal) revealed significantly higher B7-H3 protein levels in the stroke group than in controls for the 57-kDa band (*p* = 0.0006), the 53-kDa band (*p* = 0.0037), and the 34-kDa band (*p* < 0.0001) (Fig. 3C). Similarly, in aged females, two-tailed unpaired *t*-tests (with Welch’s correction applied when variances were unequal) showed significant increases in B7-H3 protein expression in the stroke groups compared with controls for the 57-kDa band (*p* = 0.0045) and for the 53- and 34-kDa bands (both *p* < 0.0001) (Fig. 3D).

Normotensive Wistar-Kyoto (WKY) rats are the standard control strain for spontaneously hypertensive rats (SHRs). Following 1-h MCAO, modified neurological severity scores (mNSS) were 7.67 ± 0.21 and 6.5 ± 0.34 at 2–4 h and on day 1 post-MCAO, respectively, in WKY rats (Fig. 3E). In contrast, SHRs exhibited markedly higher mNSS scores of 12.0 ± 0.45 and 11.33 ± 0.71 at 2–4 h and on day 1 post-MCAO, respectively, indicating that an identical duration of MCAO produced more severe neurological deficits in hypertensive rats. Similarly, 1-h MCAO in young male WKY rats and SHRs increased B7-H3 mRNA expression in the ipsilateral (ischemic) hemisphere on post-MCAO day 3 in SHRs, whereas no increase was observed in WKY rats (Fig. 3F). A two-tailed unpaired t-test (with Welch’s corrections where appropriate) confirmed that B7-H3 mRNA levels were significantly higher in stroke-induced SHRs than in their control counterparts on post-MCAO day 3 (*p* = 0.0013). Western blot analysis of ipsilateral (ischemic) hemisphere samples collected on post-MCAO day 3 from male SHRs showed increased B7-H3 protein expression in the stroke group compared with the control group (Fig. 3G). Two-tailed unpaired *t*-tests revealed significantly elevated B7-H3 protein levels in stroke-induced SHRs for the 57-kDa band (*p* = 0.0029) and for the 53- and 34-kDa bands (both *p* < 0.0001) (Fig. 3H).

### 3.4. Species, sex, age, and time after stroke influence the extent of B7-H3 degradation in the ischemic brain

**Fig. 4.**
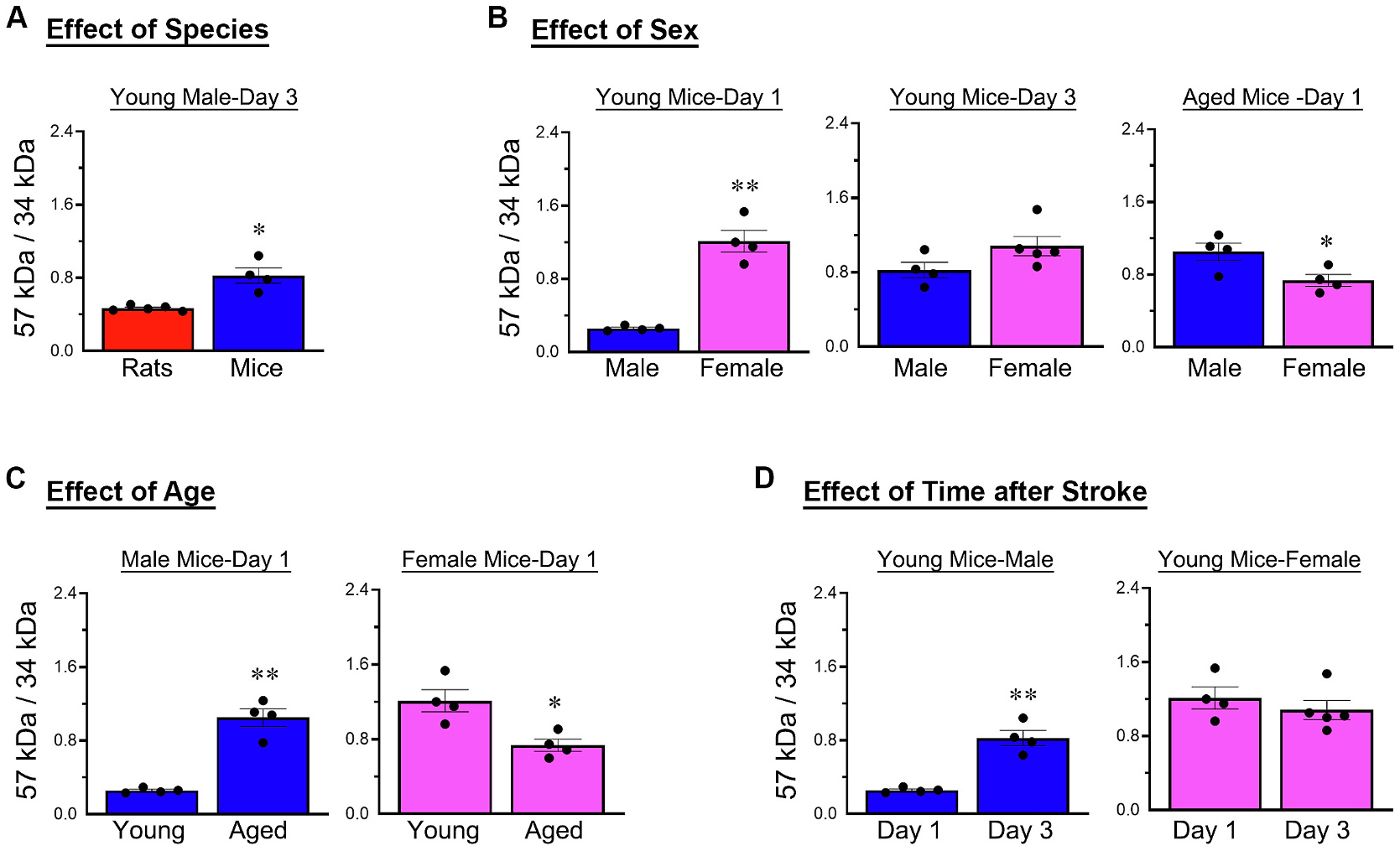
Species, sex, age, and time after stroke influence the extent of B7-H3 degradation in the ischemic brain after cerebral I/R. Column scatter plots show the effects of species (A), sex (B), age (C), and time after stroke (D) on the ratio of the 57-kDa to 34-kDa B7-H3 bands in the ipsilateral (ischemic) hemisphere. Error bars indicate SEM; n = 4–5/group. *p < 0.05, **p < 0.01 versus the corresponding experimental group.

The ratio of the 57-kDa band, representing the glycosylated full-length, membrane-bound 2IgB7-H3 isoform to the 34-kDa band, representing the putative soluble B7-H3 (sB7-H3) form that may be generated through proteolytic cleavage or degradation of the 57-kDa form, was calculated in ipsilateral (ischemic) hemisphere samples from rats and mice subjected to cerebral I/R. An increase in the 57/34-kDa ratio suggests reduced proteolytic cleavage or degradation of B7-H3 whereas a decrease in the ratio suggests enhanced cleavage or degradation. Two-tailed unpaired t-tests (with Welch’s correction applied when variances were unequal) showed that the 57/34-kDa ratio was significantly higher (1) in young male mice than in age- and sex-matched SD rats on post-MCAO day 3 (*p* = 0.0221) (Fig. 4A); (2) in young female mice than in age-matched male mice on post-MCAO day 1 (*p* = 0.0038) (Fig. 4B); (3) in aged male mice than in sex-matched young mice on post-MCAO day 1 (*p* = 0.0034) (Fig. 4C); and (4) in young male mice on post-MCAO day 3 than in age- and sex-matched mice on post-MCAO day 1 (*p* = 0.0058) (Fig. 4D). In contrast, the 57/34-kDa ratio was significantly lower in aged female mice than in age-matched males on post-MCAO day 1 (*p* = 0.0356) (Fig. 4B) and lower in aged female mice than in sex-matched young mice on post-MCAO day 1 (*p* = 0.0127) (Fig. 4C). Collectively, these findings demonstrate that species, sex, age, and time after stroke influence the extent of B7-H3 degradation in the ischemic brain following cerebral I/R.

### 3.5. Increased B7-H3 immunoreactivity is largely restricted to the ipsilateral (ischemic) hemisphere

**Fig. 5.**
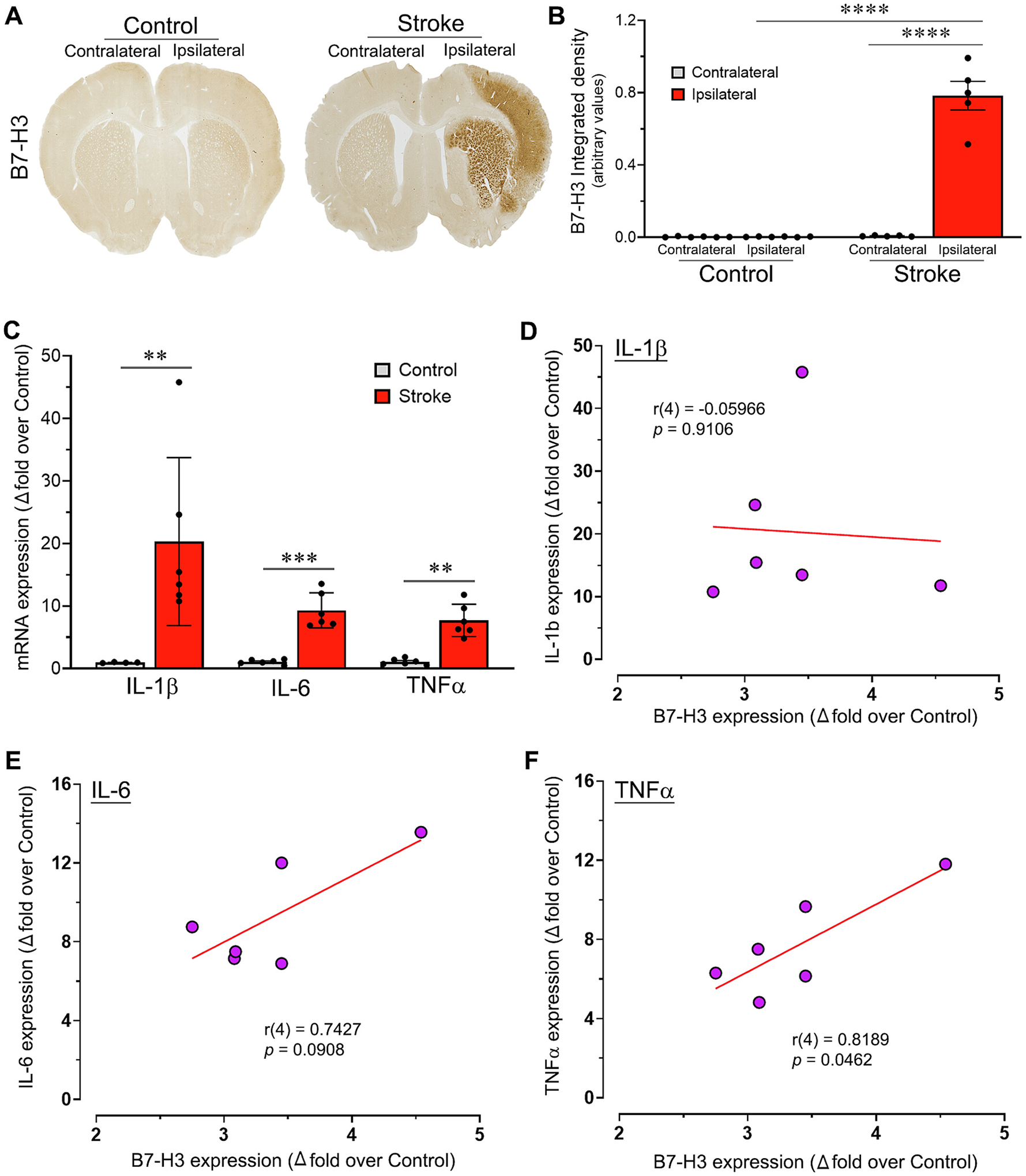
Post-stroke upregulation of B7-H3 and pro-inflammatory cytokines in the brain and the correlation between B7-H3 and cytokine expression. (A) Representative coronal brain sections from young male Sprague-Dawley rats collected on post-MCAO day 3 show B7-H3 immunohistochemistry with DAB in control animals and in rats subjected to 2-h MCAO followed by reperfusion. B7-H3 immunoreactivity is predominantly localized to the ipsilateral (ischemic) hemisphere, particularly in the somatosensory cortex and striatum, primary regions affected in this MCAO model. (B) Column scatter plot shows the quantified integrated density of B7-H3 staining in the contralateral (non-ischemic) and ipsilateral (ischemic) hemispheres of control and stroke groups. Error bars indicate SEM; n = 5–6/group. ****p < 0.0001. (C) Column scatter plot shows mRNA expression of IL-1β, IL-6, and TNFα in the ipsilateral (ischemic) hemispheres of rats on post-MCAO day 3. Error bars indicate SEM; n = 4–6/group. **p < 0.01; ***p < 0.001. (D–_F)_ Scatter plots show the relationships between B7-H3 mRNA expression and mRNA expression of IL-1β (D), IL-6 (E), and TNFα (F) in the ipsilateral (ischemic) hemisphere on post-MCAO day 3 in stroke-induced rats. A significant positive correlation is observed between B7-H3 and TNFα, whereas correlations with IL-1β and IL-6 do not reach statistical significance.

Immunohistochemical analysis of coronal brain sections from SD rats collected on post-MCAO day 3 revealed prominent B7-H3 immunoreactivity (Fig. 5A). In control animals, B7-H3 staining was minimal in both hemispheres. In contrast, stroke animals exhibited robust B7-H3 staining that was predominantly confined to the ipsilateral (ischemic) hemisphere, particularly in the somatosensory cortex and caudate putamen (striatum), the primary regions affected by MCAO. In preliminary optimization experiments, brain sections were processed in parallel either with the B7-H3 primary antibody or without the primary antibody (negative control) and carried through secondary antibody incubation and DAB development. No detectable staining in the cortex or striatum was observed in the negative control sections, supporting the specificity of B7-H3 immunostaining. Quantitative analysis showed that the B7-H3-positive area comprised approximately 23% of the ipsilateral (ischemic) hemisphere in the stroke group versus 0.1% in the control group (Fig. 5B). These findings suggest that B7-H3 upregulation is specific to ischemic injury and not attributable to surgical manipulation or procedural stress. Two-way ANOVA with Sidak’s multiple comparisons test showed that B7-H3 immunoreactivity, quantified as integrated density, was significantly greater in the ipsilateral (ischemic) than in the contralateral hemisphere within the stroke group, and was also significantly greater in the ipsilateral (ischemic) hemisphere of stroke animals than in the ipsilateral hemisphere of control animals (*p* < 0.0001 for both comparisons).

### 3.6. B7-H3 expression exhibits a positive correlation with TNFα expression

Cerebral I/R induced by MCAO in young male SD rats increased mRNA expression of the pro-inflammatory cytokines IL-1β, IL-6, and TNFα in the ipsilateral (ischemic) hemisphere on post-MCAO day 3 (Fig. 5C). Cytokine expression was significantly elevated in the stroke group compared with the control group for IL-1β (*p* = 0.0095; Mann-Whitney test), IL-6 (*p* = 0.0007; two-tailed unpaired *t*-test with Welch’s correction), and TNFα (*p* = 0.0013; two-tailed unpaired t-test with Welch’s correction). We next examined the relationships between B7-H3 mRNA expression and the mRNA expression of these pro-inflammatory cytokines on post-MCAO day 3 in stroke-induced SD rats. B7-H3 expression showed positive correlations with IL-6 and TNFα, but not with IL-1β (Fig. 5D–F). However, the correlation was statistically significant only between B7-H3 and TNFα expression (Pearson’s r(4) = 0.8189, *p* = 0.0462), indicating that higher B7-H3 expression is associated with higher TNFα expression in the ischemic brain.

## 4. Discussion

To our knowledge, this study is the first to demonstrate that B7-H3 expression is upregulated in the ischemic brain after cerebral ischemia/reperfusion (I/R) in two rodent species, encompassing males and females, young and aged animals, and animals with hypertension, a prevalent comorbidity among stroke patients. B7-H3 upregulation was largely confined to the ipsilateral (ischemic) hemisphere and was positively correlated with the expression of the pro-inflammatory cytokine TNFα. In young male SD rats, B7-H3 mRNA levels increased over the first week after ischemic stroke, peaking on post-MCAO day 3 and paralleling the time window associated with secondary ischemic injury. Moreover, B7-H3 protein levels were significantly elevated in the ipsilateral (ischemic) hemisphere at this time point, consistent with peak mRNA expression. Although the temporal mRNA profile in young male SD rats showed statistically significant increases primarily on post-MCAO days 3 and 5, upregulation of B7-H3-immunoreactive bands was evident as early as post-MCAO day 1 in both sexes of young and aged mice, indicating that B7-H3 protein induction can occur early after ischemic injury in this model. This difference may reflect variations in experimental parameters between models, including differences in MCAO duration and stroke severity. To place these temporal changes in the context of neuroinflammatory signaling, we found that mRNA levels of IL-1β, IL-6, and TNFα were significantly increased in the ipsilateral (ischemic) hemisphere on post-MCAO day 3. Notably, B7-H3 mRNA expression was significantly and positively correlated with TNFα mRNA expression, whereas no significant correlations were observed with IL-1β or IL-6.

Immune checkpoints regulate the onset, severity, and duration of immune responses by mediating activating and inhibitory signals (Carlino et al., 2021). Among the 10 members of the B7 superfamily of immune checkpoints, B7-H3, a 316-amino-acid type 1 transmembrane protein, has attracted considerable interest since its discovery in 2001 (Chapoval et al., 2001; Liu et al., 2021). Although soluble B7-H3 is known to bind CD4+ T cells, CD8+ T cells, natural killer (NK) cells, and natural killer T (NKT) cells, the specific receptor for B7-H3 remains unidentified (Kontos et al., 2021). Due to alternative splicing and post-translational modifications (PTMs) such as glycosylation, B7-H3 exists in multiple forms with different molecular weights. In humans, the main isoforms of B7-H3 include the full-length membrane-associated 4Ig-B7-H3 (~45–66 kDa), the alternatively spliced 2Ig-B7-H3 variant (~34–57 kDa), and a soluble form, sB7-H3 (~37 kDa). While the 2Ig-B7-H3 isoform contains a single pair of extracellular V- and C-like Ig domains, a transmembrane region, and a cytoplasmic tail, the dominantly expressed human 4Ig-B7-H3 isoform contains two identical pairs of V- and C-like Ig domains (Chapoval et al., 2001; Sun et al., 2002). Previous work has shown that the 4Ig-B7-H3 isoform suppresses cytokine expression and inhibits T-cell proliferation, whereas the 2Ig-B7-H3 isoform enhances T-cell proliferation and upregulates IL-2 and IFNγ expression (Sun et al., 2011). Within the brain, cerebral microvascular endothelial cells may express low levels of B7-H3 mRNA; however, B7-H3 protein expression is minimal or undetectable under normal conditions (Digregorio et al., 2021). Because our immunoblotting and real-time PCR analyses were performed on whole ischemic brain homogenates, these assays reflect tissue-level changes and do not resolve the specific B7-H3-expressing cell type(s). Based on the available literature and the neurovascular response to cerebral I/R, cerebral microvascular endothelial cells may represent an important source of B7-H3 in the ischemic brain; however, additional brain cell types may also contribute. Therefore, future studies should focus on identifying the cellular sources of B7-H3 in the ischemic brain.

In this study, three predominant B7-H3-immunoreactive bands were detected by Western blot analysis in ipsilateral (ischemic) hemisphere samples from both rats and mice subjected to cerebral I/R. The bands observed in Western blots had approximate molecular weights of 57, 53, and 34 kDa. It has been reported that human B7-H3 comprises two isoforms (2Ig-B7-H3 and 4Ig-B7-H3), whereas murine B7-H3 exists as a single isoform (2Ig-B7-H3) (Ling et al., 2003; Sun et al., 2011). The 57-, 53-, and 34-kDa bands detected in the ischemic brains of rats and mice may reflect differences in PTMs and/or proteolytic processing of B7-H3. PTMs enhance the functional diversity of proteins, can affect normal cellular function, and may contribute to disease pathogenesis (Getu et al., 2023). The best-characterized PTM of B7-H3 is glycosylation. During glycosylation, carbohydrate chains are covalently attached to the asparagine residues of B7-H3, increasing its apparent molecular weight. Accordingly, the reported molecular weights of the glycosylated 4Ig-B7-H3 and 2Ig-B7-H3 isoforms in humans are approximately 90–100 kDa and 50–70 kDa, respectively. In mice, the calculated molecular weight of the full-length 2Ig-B7-H3 isoform is approximately 25–28 kDa. Thus, the 57-kDa band observed in the ipsilateral (ischemic) hemispheres of rodents may correspond to the glycosylated, full-length, membrane-associated 2Ig-B7-H3 isoform, whereas the 53-kDa band may represent a less glycosylated and/or otherwise processed form. The 34-kDa band may correspond to soluble B7-H3 (sB7-H3), potentially arising from proteolytic cleavage of the extracellular domain of membrane-associated B7-H3 and/or alternative mRNA processing (Sun et al., 2011). Although the contribution of alternative mRNA splicing to sB7-H3 generation remains unclear, cleavage of the extracellular domain of membrane-bound, glycosylated 2Ig-B7-H3 by matrix metalloproteinases (MMPs) has been recognized as a primary mechanism underlying sB7-H3 formation (Zhang et al., 2008). If sB7-H3 levels increase in serum after ischemic stroke, sB7-H3 could serve as a clinically valuable biomarker for assessing stroke severity, neuroinflammatory burden, or therapeutic response.

B7-H3 glycosylation has been reported to be important for its function (Huang et al., 2021). Accordingly, B7-H3-immunoreactive bands at 57 kDa and 53 kDa likely represent mature, glycosylated forms and are the most plausible candidates for functional, membrane-bound B7-H3. By contrast, the 34-kDa band may reflect a soluble, cleaved, or otherwise processed form of B7-H3, and its functional activity in the post-ischemic brain remains to be determined.

To evaluate putative proteolytic processing of B7-H3 across experimental groups, we calculated the 57/34-kDa ratio as an index of B7-H3 degradation. This analysis indicated that species, sex, age, and time post-stroke are associated with differences in B7-H3 processing in the ischemic brain after cerebral I/R. As described above, an increased 57/34-kDa ratio suggests reduced proteolytic cleavage or degradation of B7-H3, whereas a decreased ratio suggests enhanced degradation. Across groups, B7-H3 degradation was lower in mice than in rats. Among young mice on post-MCAO day 1, females exhibited less degradation (higher 57/34-kDa ratios) than males. In contrast, among aged mice on post-MCAO day 1, females showed greater degradation than males. When age groups were compared within sex on post-MCAO day 1, aged males displayed less degradation than young males, whereas aged females showed greater degradation than young females. Finally, in young male mice, B7-H3 degradation was lower on post-MCAO day 3 than on post-MCAO day 1. Although these variations were observed across species, sex, age, and post-MCAO time points, the factors driving these differences cannot be determined from the present study. Nevertheless, our previous work showing distinct temporal patterns of MMP expression in the ischemic brain, together with evidence that MMPs can cleave membrane-bound B7-H3 to generate soluble B7-H3, suggests that MMP-dependent proteolytic processing may contribute to the observed banding patterns and 57/34-kDa ratios (Zhang et al., 2008; Chelluboina et al., 2015). This possibility was not directly tested in the present study. Future studies are needed to determine whether levels of B7-H3-immunoreactive bands and/or 57/34-kDa ratios correlate with stroke severity (e.g., mNSS) across the experimental groups examined.

Hypertension is a significant risk factor for ischemic stroke and, as a comorbid condition, increases both the incidence and severity of stroke (Fisher et al., 2007; Howells et al., 2010; O’Donnell et al., 2010). The SHR is the most widely used genetic model of hypertension. Compared with normotensive wild-type WKY rats, SHRs exhibit consistently elevated blood pressure beginning at approximately 4 weeks of age (Dickhout and Lee, 1998). In this study, 3-month-old SHRs were subjected to cerebral I/R. Under our experimental conditions, 1-h MCAO produced more severe neurological deficits in SHRs than in normotensive WKY rats, as reflected by mNSS values, illustrating the impact of hypertension on stroke severity after cerebral I/R. Moreover, stroke severity, as measured by mNSS, was greater in SHRs subjected to 1-h MCAO than in normotensive SD rats subjected to 2-h MCAO. As expected, B7-H3 mRNA expression was significantly increased in SHRs compared with WKY rats. In addition, protein expression levels of all B7-H3-immunoreactive bands were markedly elevated in stroke-induced SHRs relative to control SHRs. Consistent with our findings in SD rats, the expression level of the 57-kDa B7-H3 band in SHRs on post-MCAO day 3 was lower than that of the 53- and 34-kDa bands.

The robust B7-H3 immunoreactivity observed in the cortex and striatum of the ipsilateral (ischemic) hemisphere, the primary regions impacted by MCAO, together with undetectable or very low levels of B7-H3 immunoreactivity in the contralateral hemisphere, suggests a potential role for B7-H3 in stroke pathology. Increased B7-H3 expression in the ipsilateral (ischemic) hemisphere after cerebral I/R was positively correlated with the expression of the key pro-inflammatory cytokine TNFα, suggesting that B7-H3 may participate in the inflammatory cascade after cerebral I/R. This finding is consistent with prior evidence that B7-H3 can promote the release of pro-inflammatory cytokines from monocytes and macrophages (Zhang et al., 2010). Thus, our data provide evidence that B7-H3 is not only upregulated in the ischemic brain but may also be linked to pro-inflammatory cytokine signaling. However, these observations do not establish whether elevated B7-H3 expression in the ischemic brain is ultimately harmful or beneficial. The correlation between B7-H3 and TNFα after cerebral I/R may also indicate that B7-H3 expression increases to protect brain cells from excessive neuroinflammation. For example, in the tumor microenvironment, B7-H3 promotes macrophage polarization from an M1 (pro-inflammatory) to an M2 (anti-inflammatory) phenotype, contributing to an immunosuppressive microenvironment through the CCL2-CCR2-M2 macrophage axis. B7-H3 further enhances immunosuppression by increasing IL-10 and TGFβ production, inhibiting the activity of multiple T-cell subsets and other immune cells, and reducing the secretion of IFNγ, IL-2, perforin, and granzyme B (Mao et al., 2017; Han et al., 2018; Lu et al., 2020; Lee et al., 2021; Long et al., 2021; Si et al., 2022; Miyamoto et al., 2022). Although B7-H3 is recognized as an immuno-regulatory molecule with context-dependent co-stimulatory or coinhibitory functions, its role in neuroinflammation after cerebral I/R remains unknown. In the ischemic microenvironment, B7-H3 may therefore act either as a friend or a foe.

In summary, B7-H3 expression was significantly upregulated in the ischemic brains of young rats and in both young and aged male and female mice. B7-H3 immunoreactivity was largely confined to the ipsilateral (ischemic) hemisphere and was particularly prominent in the cortex and striatum, the primary regions affected by MCAO, suggesting a role in stroke pathology. Although B7-H3 is best known for its role in immune regulation, its increased expression after stroke highlights its potential as a therapeutic target. The specific role of B7-H3 in neuroinflammation and brain damage after cerebral I/R, however, remains unknown. Elucidating the function of B7-H3 in the ischemic brain microenvironment may lead to the discovery of novel therapeutic targets and the development of treatments that reduce brain damage and enhance functional recovery after cerebral I/R. In this study, B7-H3 expression in the ischemic brains of rats was positively correlated with expression of the pro-inflammatory cytokine TNFα, raising the possibility that blocking B7-H3 could mitigate post-ischemic neuroinflammation, a key contributor to secondary brain injury. B7-H3 may also serve as a marker of neuroinflammatory burden in the brain after cerebral I/R. The scope of this study was limited to demonstrating B7-H3 upregulation in the brain after cerebral I/R in preclinical rodent models. Therefore, future studies will focus on identifying the cellular sources of B7-H3 in the ischemic brain and characterizing its role in post-stroke pathogenesis, particularly neuroinflammation, brain damage, and recovery of sensorimotor and cognitive functions. These studies will incorporate genetic and pharmacological approaches in aged animals and in animals with comorbidities to better reflect the heterogeneous health status of the acute ischemic stroke patient population.

## Supplementary Material

1

## Data Availability

All data supporting the key findings of this study, including the statistical tests employed and exact *p* values, are included in the article. The original uncropped digital images of the Western blots, images of all brain sections processed by immunohistochemistry, and ImageJ analysis data are provided as supplementary data. Additional raw real-time PCR data, including instrument-generated files and associated analyses, are available from the corresponding author upon reasonable request.

## Appendix A. Supplementary data

Supplementary data to this article can be found online at https://doi.org/10.1016/j.expneurol.2026.115675.
