# Supplementary material for: B7-H3 upregulation in ischemic stroke: friend or foe?
Source: Exp Neurol. Author manuscript; Available in PMC 2026 Jul 1. (PMC13318485; doi:10.1016/j.expneurol.2026.115675)
Supplement: 1 [file NIHMS2190227-supplement-1.pdf]

Supplementary Table 1. Experimental groups, description, and animal numbers.

Group #	Group description	Number of animals		
		Included	Excluded	Total
Control groups				
R1	Young male SD rats subjected to MCAO surgical procedure without monofilament insertion and euthanized on days 3 and 7 post-surgery (for qPCR, WB, and IHC).	17	0	17
R3	Young male WKY rats subjected to MCAO surgical procedure without monofilament insertion and euthanized on day 3 post-surgery (for qPCR).	6	0	6
R5	Young male SHRs subjected to MCAO surgical procedure without monofilament insertion and euthanized on day 3 post-surgery (for qPCR and WB).	10	0	10
M1	Young male mice subjected to MCAO surgical procedure without monofilament insertion and euthanized on days 1 and 3 post-surgery (for WB).	8	0	8
M3	Young female mice subjected to MCAO surgical procedure without monofilament insertion and euthanized on days 1 and 3 post-surgery (for WB).	8	0	8
M5	Aged male mice subjected to MCAO surgical procedure without monofilament insertion and euthanized on day 1 post-surgery (for WB).	4	0	4
M7	Aged female mice subjected to MCAO surgical procedure without monofilament insertion and euthanized on day 1 post-surgery (for WB).	4	0	4
Stroke groups				
R2	Young male SD rats subjected to 2-h MCAO and euthanized on days 1, 3, 5, and 7 post-MCAO (for qPCR, WB, and IHC).	35	7	42
R4	Young male WKY rats subjected to 1-h MCAO and euthanized on day 3 post-MCAO (for qPCR).	6	2	8
R6	Young male SHRs subjected to 1-h MCAO and euthanized on day 3 post-MCAO (for qPCR and WB).	11	3	14
M2	Young male mice subjected to 1-h MCAO and euthanized on days 1 and 3 post-MCAO (for WB).	8	3	11
M4	Young female mice subjected to 1-h MCAO and euthanized on days 1 and 3 post-MCAO (for WB).	9	3	12
M6	Aged male mice subjected to 1-h MCAO and euthanized on day 1 post-MCAO (for WB).	4	2	6
M8	Aged female mice subjected to 1-h MCAO and euthanized on day 1 post-MCAO (for WB).	4	2	6

SD, Sprague-Dawley; WKY, Wistar-Kyoto; SHRs, spontaneously hypertensive rats; mNSS, modified neurological severity score; NDS, neurological deficit score; MCAO, middle cerebral artery occlusion; qPCR, quantitative real time PCR; WB, Western blot; IHC, immunohistochemistry

Supplementary Table 2. Rat primers used for real-time PCR analysis.

**Gene**	**NCBI Reference Sequence**	**Primer Sequence**	
		**Forward (5' - 3')**	**Reverse (5' - 3')**
B7-H3	NM_182824	agctgacagacaccaaaca	caccaacaagtcagggaaga
IL-1β	NM_031512	gctttcgacagtgaggagaat	cgagatgctgctgtgagatt
IL-6	NM_012589	ccaacttccaatgctctcctaa	ttgccgagtagacctcatagt
TNFα	NM_012675	cagccgatttgccatttcatac	aggtacatgggctcatacca
18S rRNA	NR_046237	acgtctgccctatcaactttc	ttggatgtggtagccgtttc

PCR, polymerase chain reaction; IL–interleukin; TNF, tumor necrosis factor

Supplementary Data – Appendix A

Raw Data of Western Blots

(Original Uncropped Digital Images)

Figure 1B

B7-H3

Bright Field Image | Chemiluminescent Image | Inverted + Desaturated

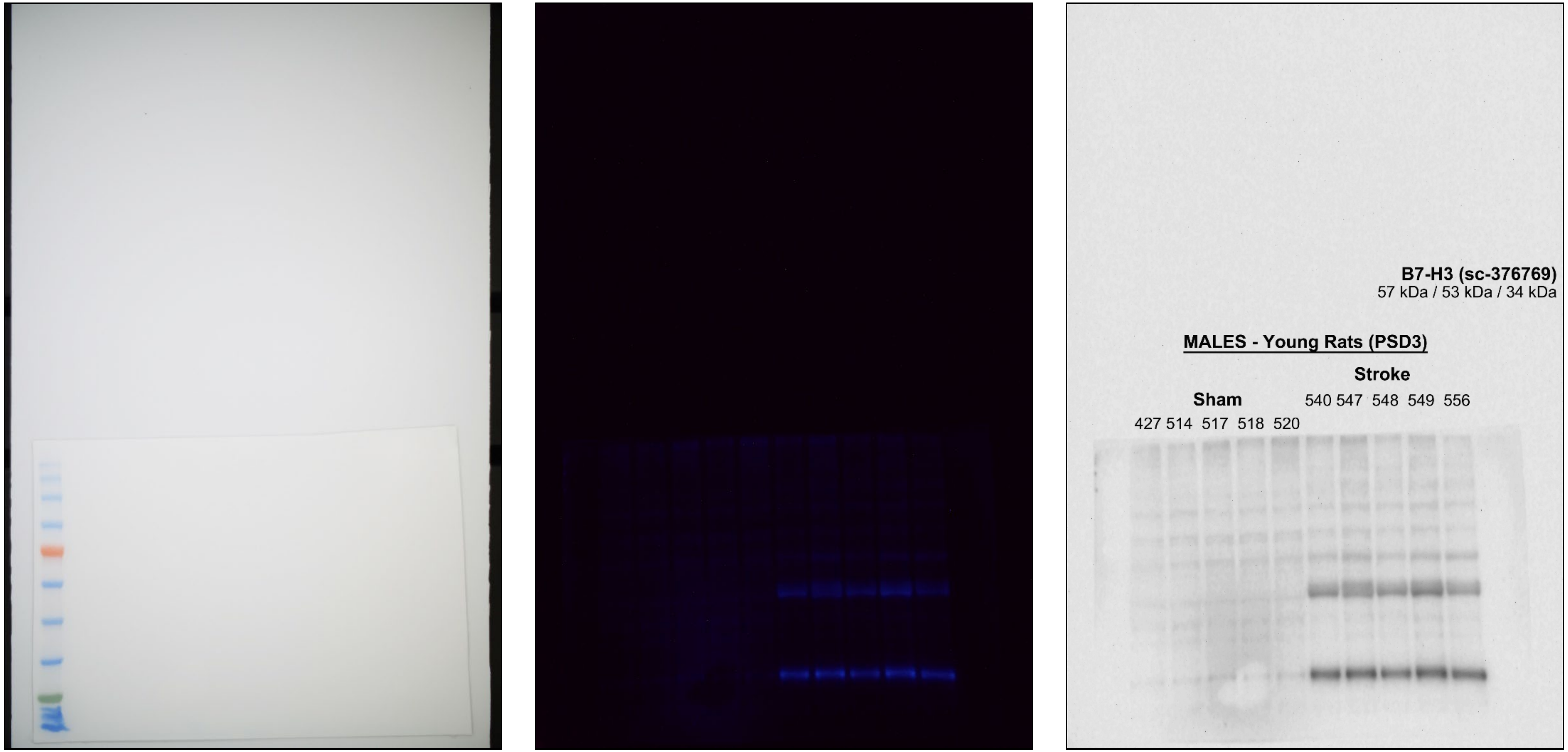

Figure 1B

B7-H3 blot Reprobed with GAPDH

Bright Field Image

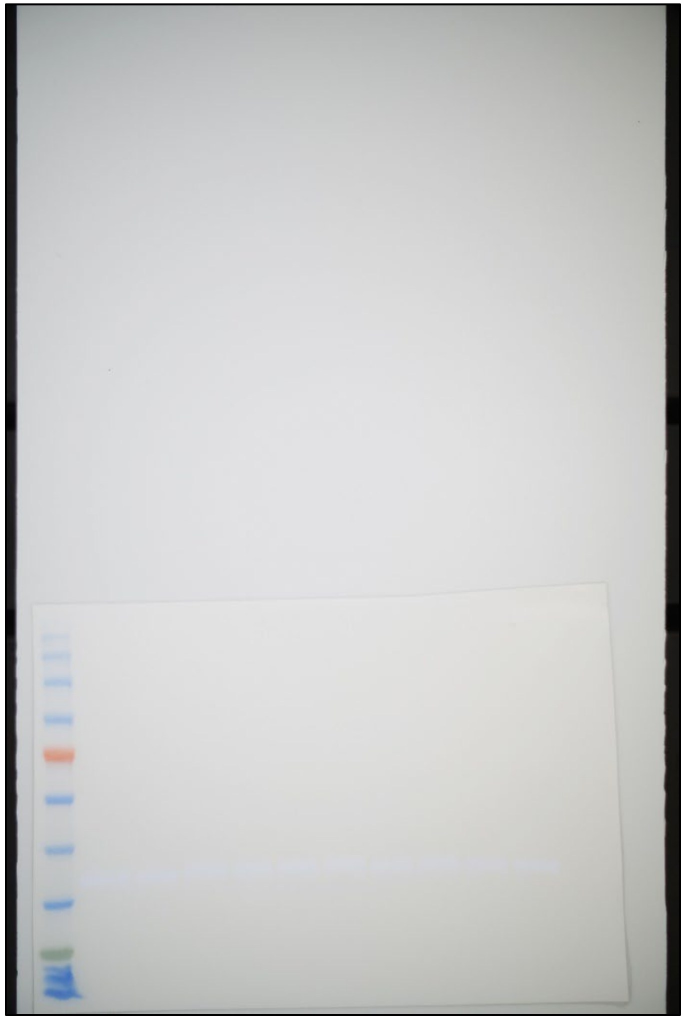

Chemiluminescent Image

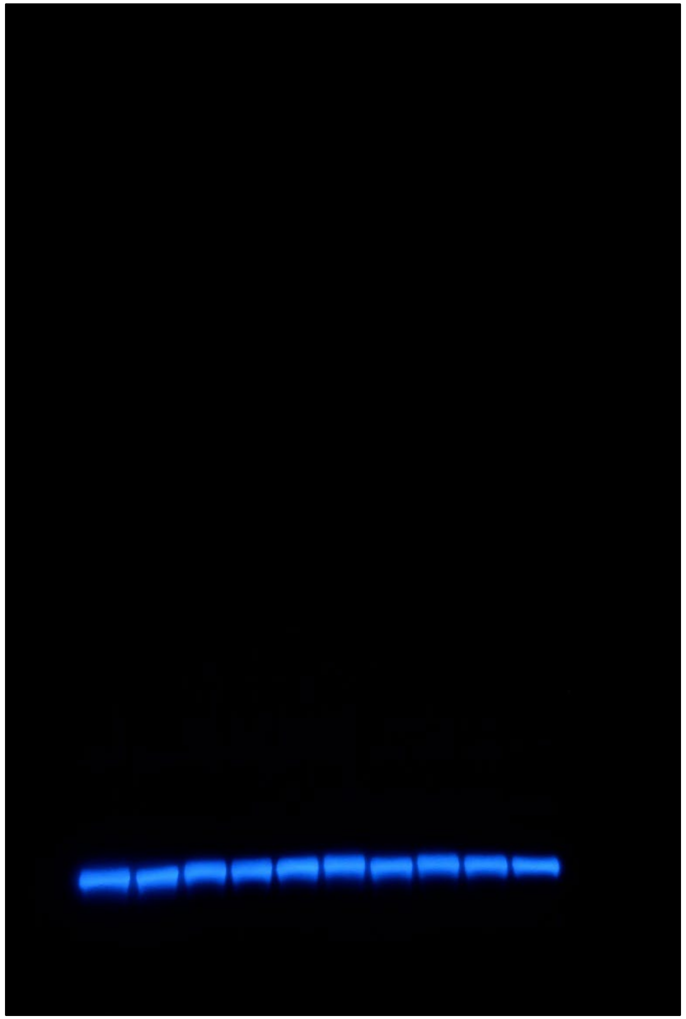

Inverted + Desaturated

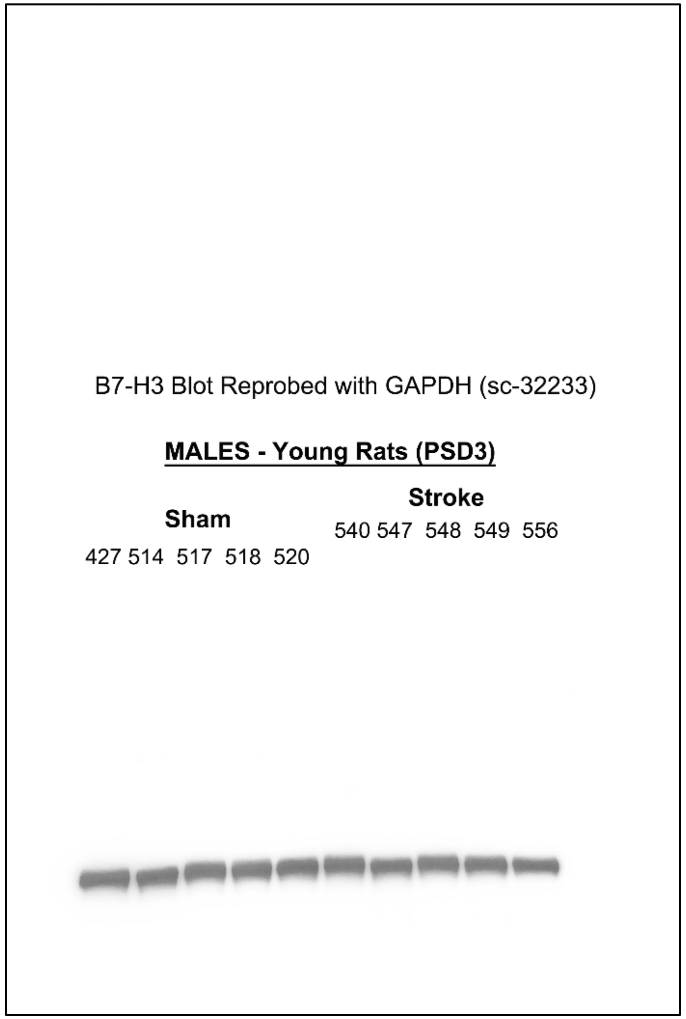

Figure 2A; Day-1

B7-H3

Bright Field Image

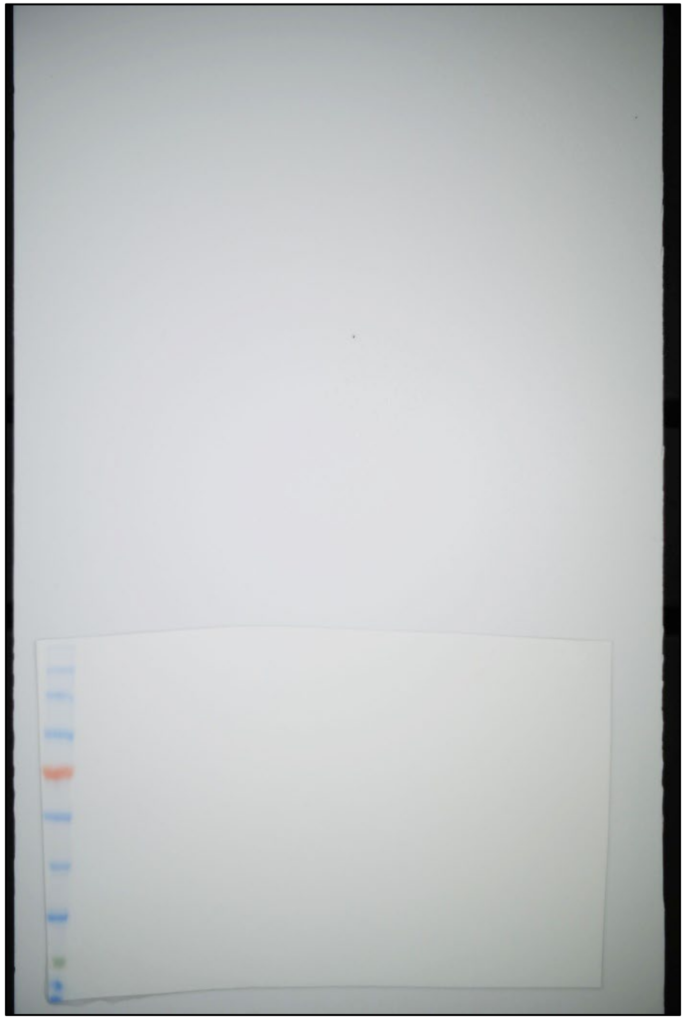

Chemiluminescent Image

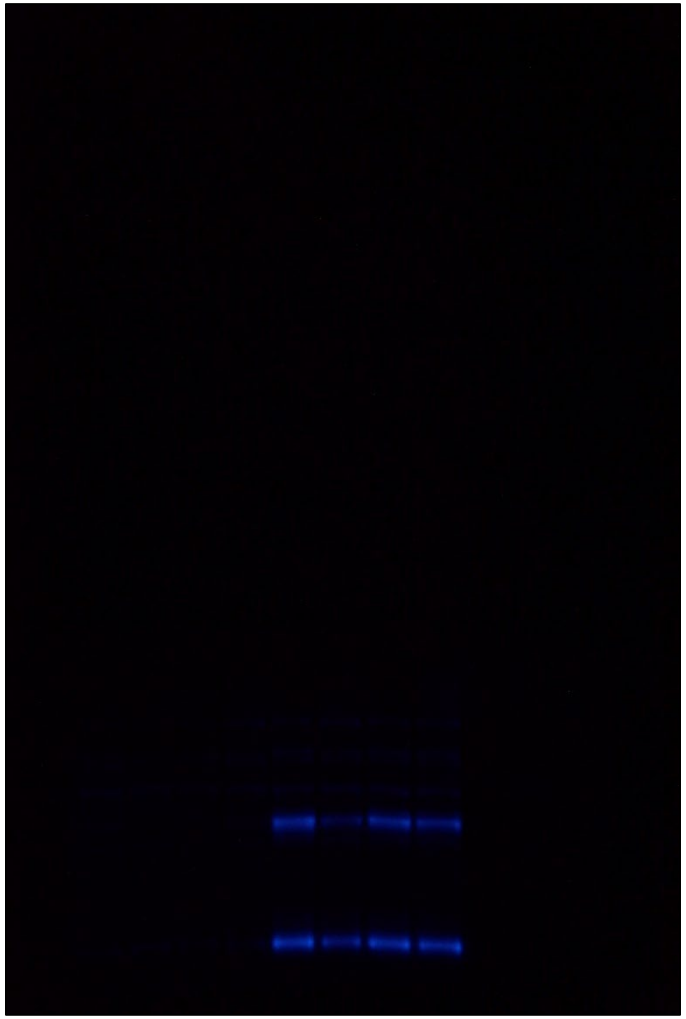

Inverted + Desaturated

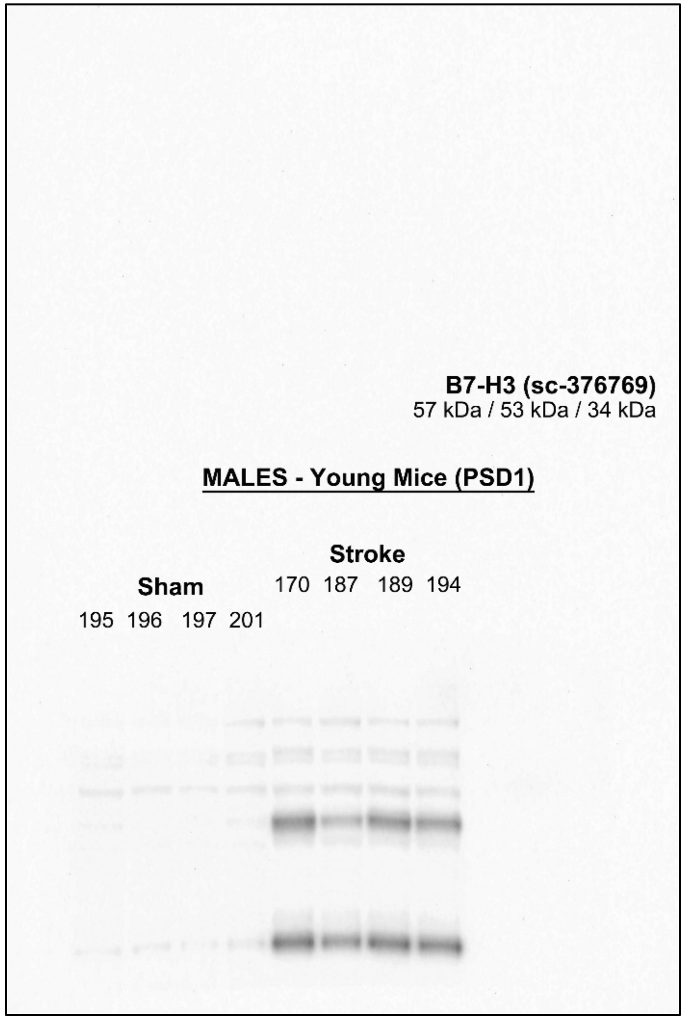

Figure 2A; Day-1

B7-H3 blot Reprobed with GAPDH

Bright Field Image

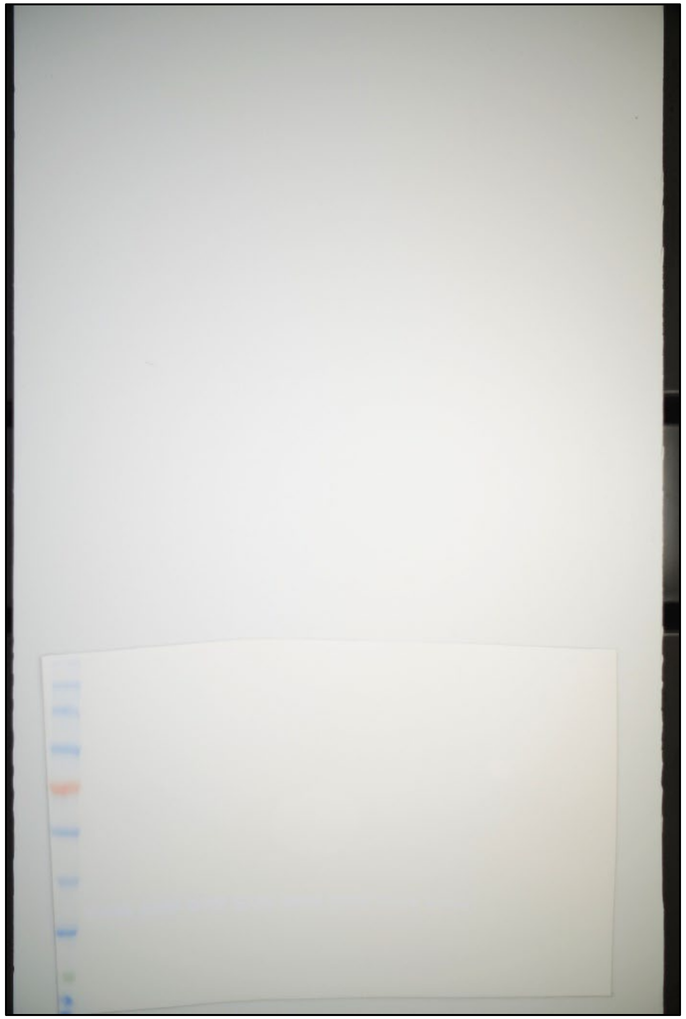

Chemiluminescent Image

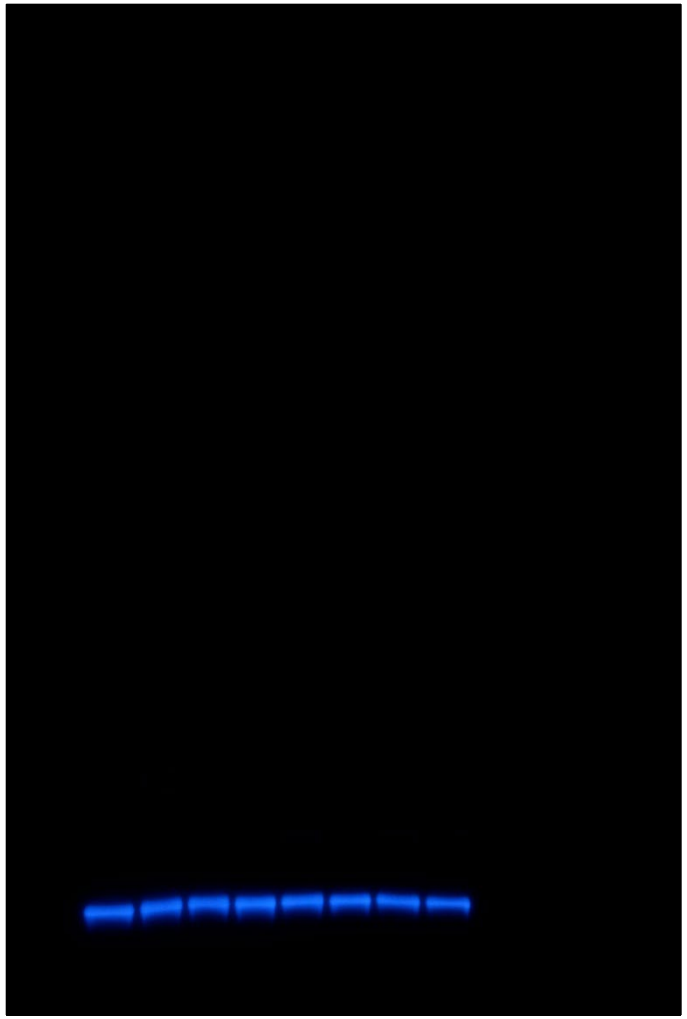

Inverted + Desaturated

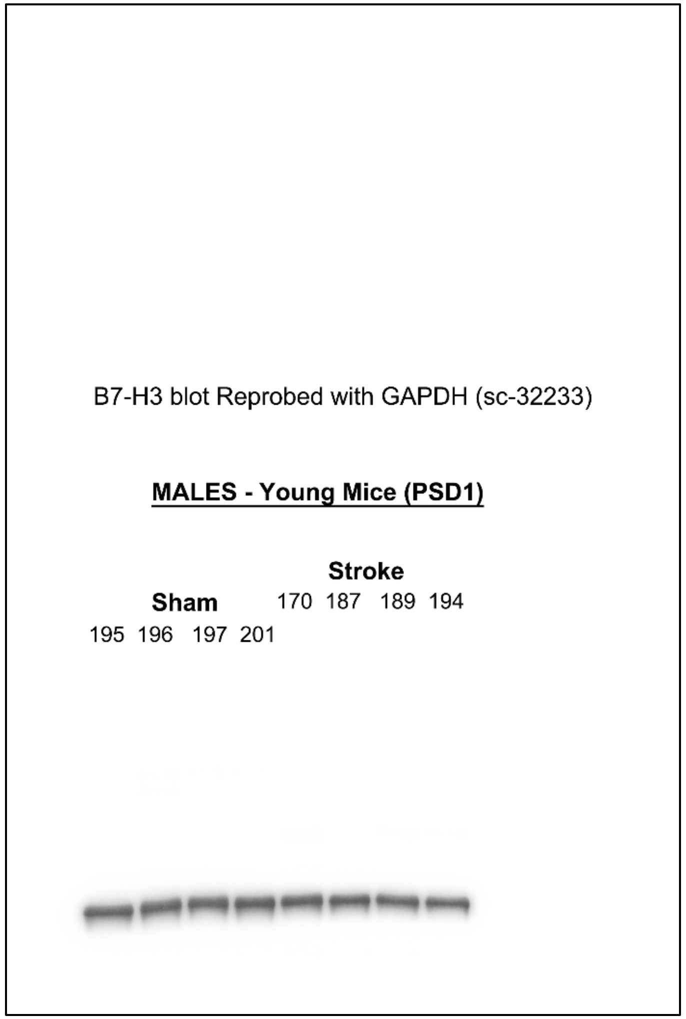

B7-H3

Figure 2A; Day-3

Bright Field Image

Chemiluminescent Image

Inverted + Desaturated

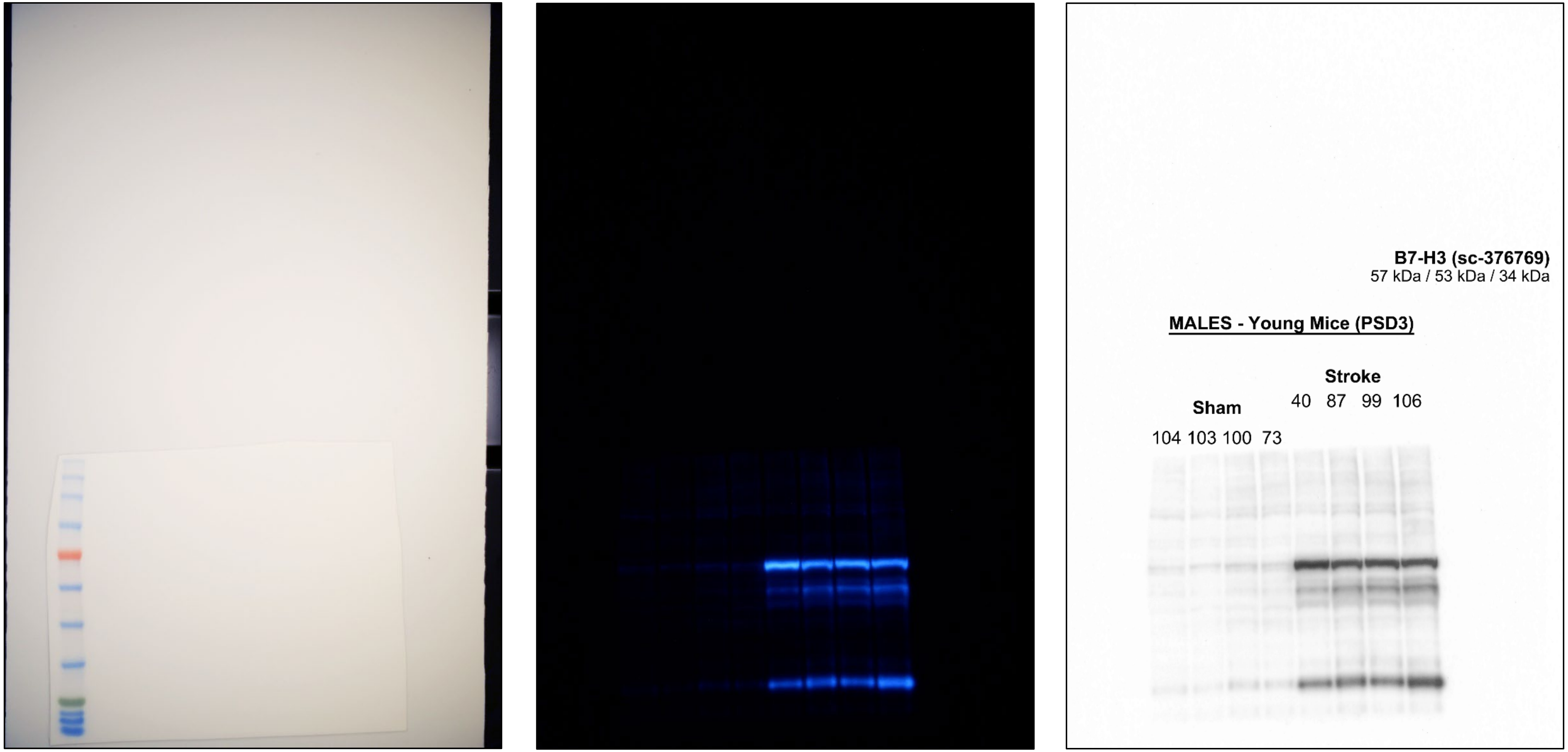

Figure 2A; Day-3

B7-H3 blot Reprobed with GAPDH

Bright Field Image

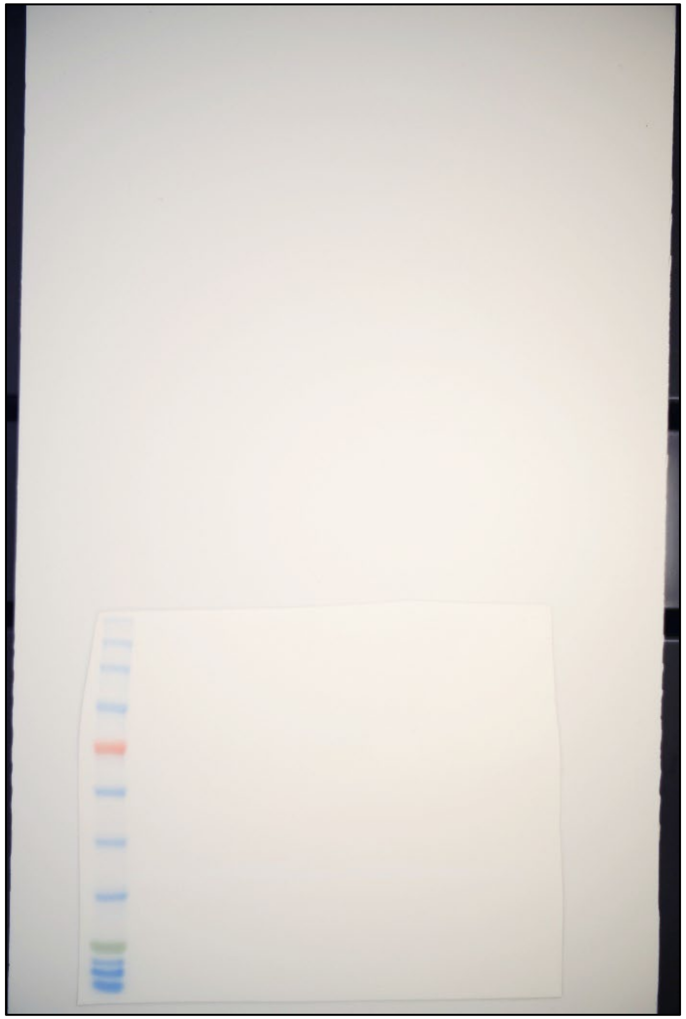

Chemiluminescent Image

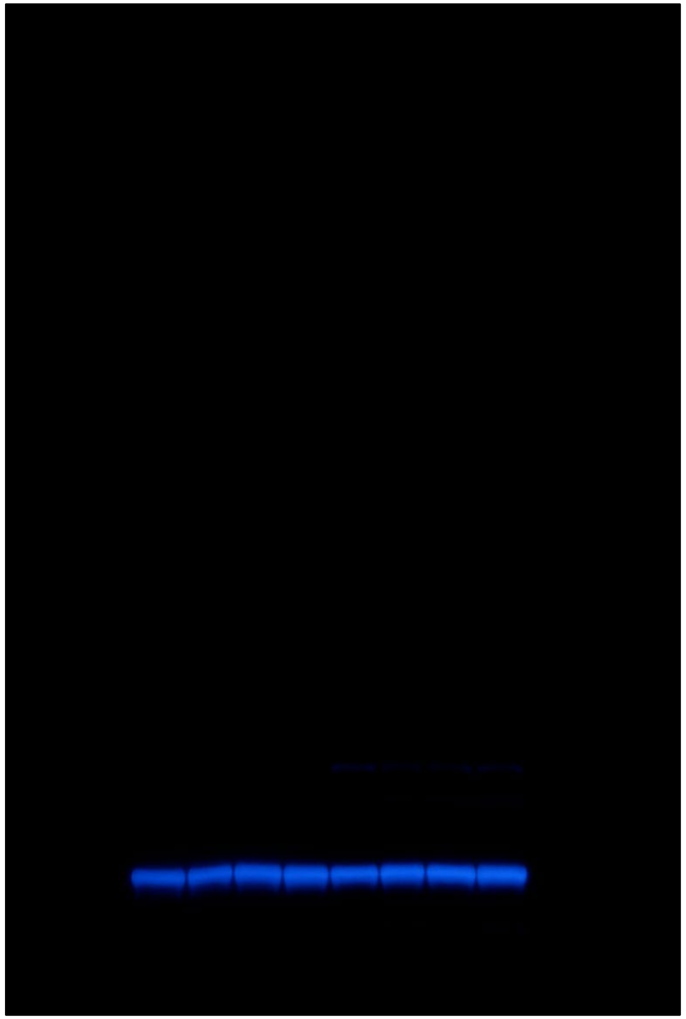

Inverted + Desaturated

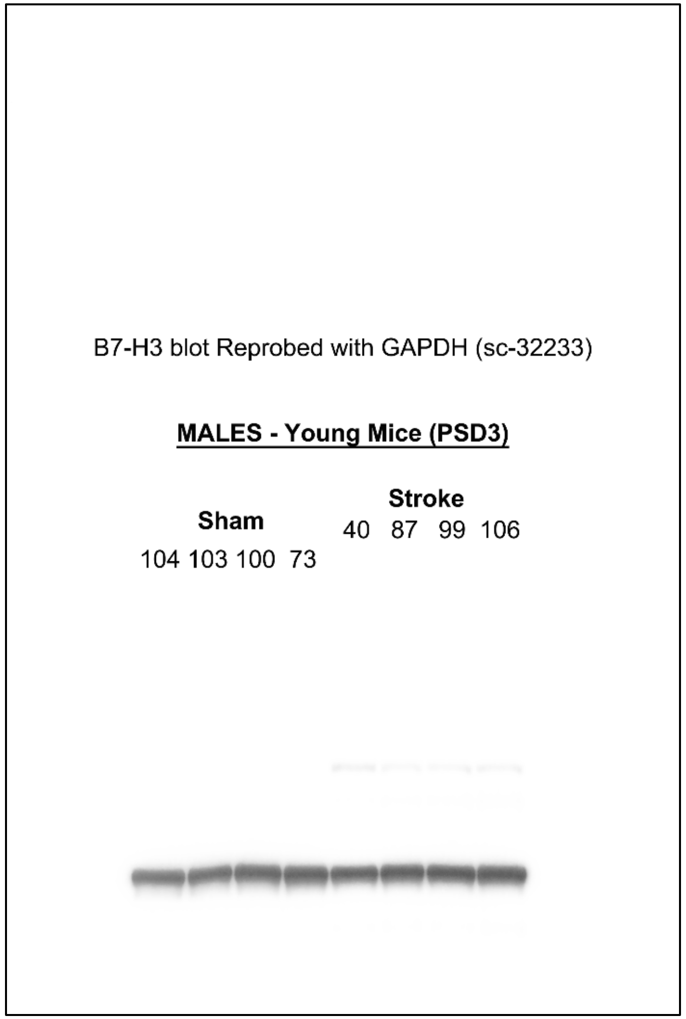

B7-H3

Figure 2B; Day-1

Bright Field Image

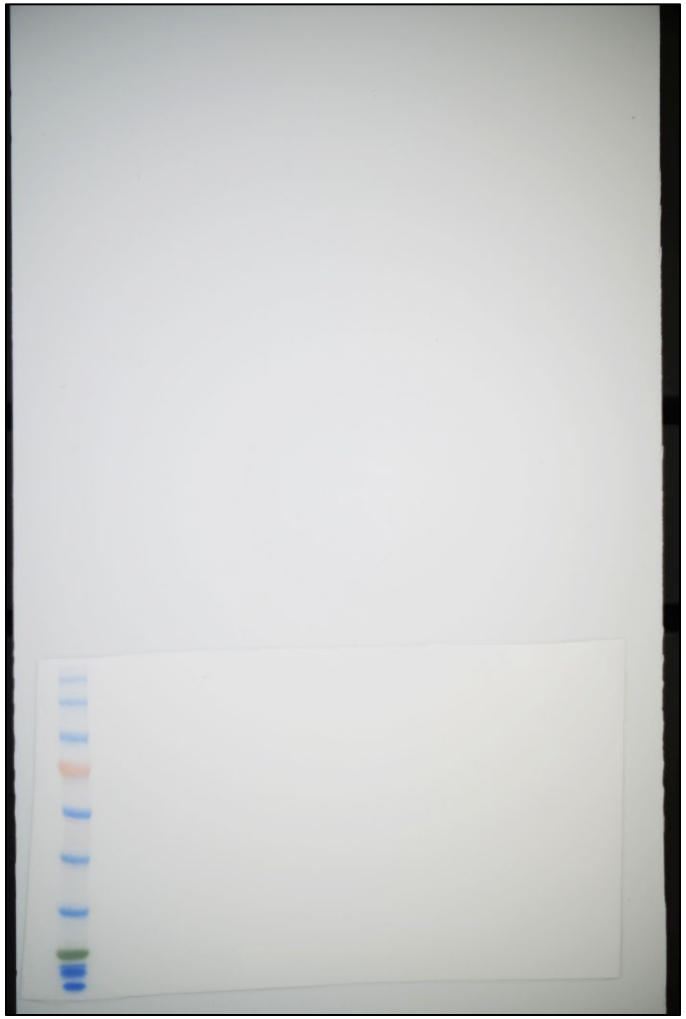

Chemiluminescent Image

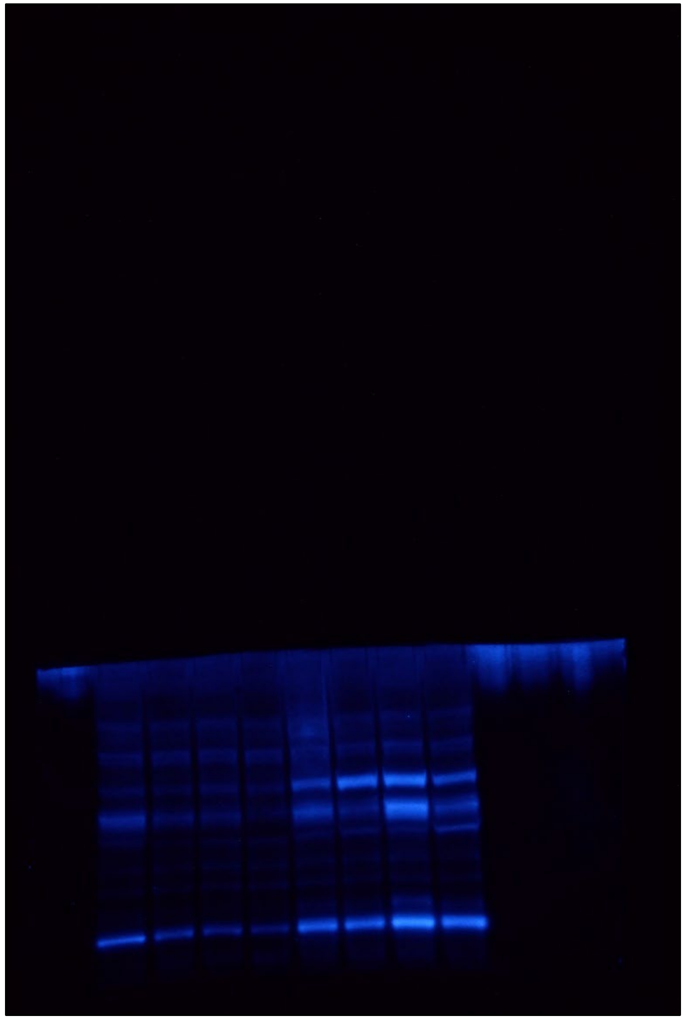

Inverted + Desaturated

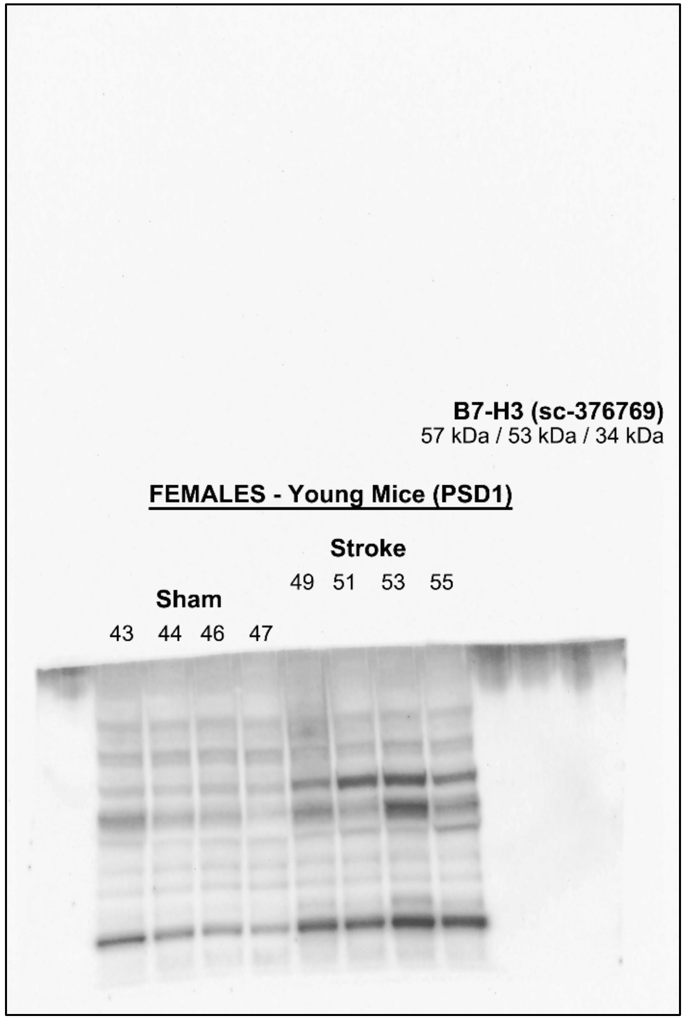

Figure 2B; Day-1

B7-H3 blot Reprobed with GAPDH

Bright Field Image

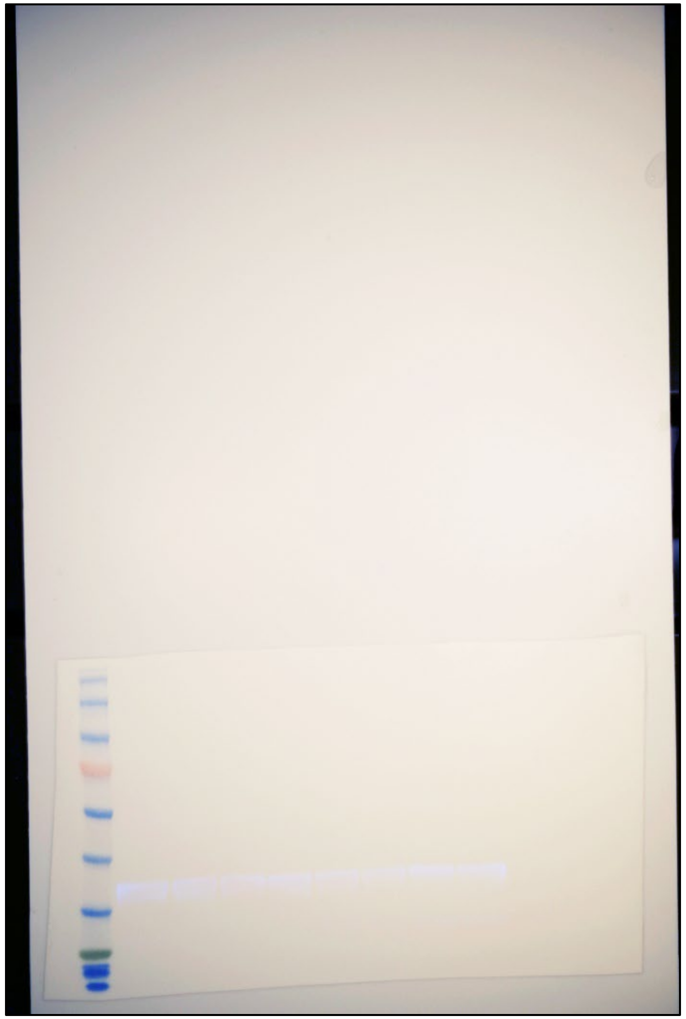

Chemiluminescent Image

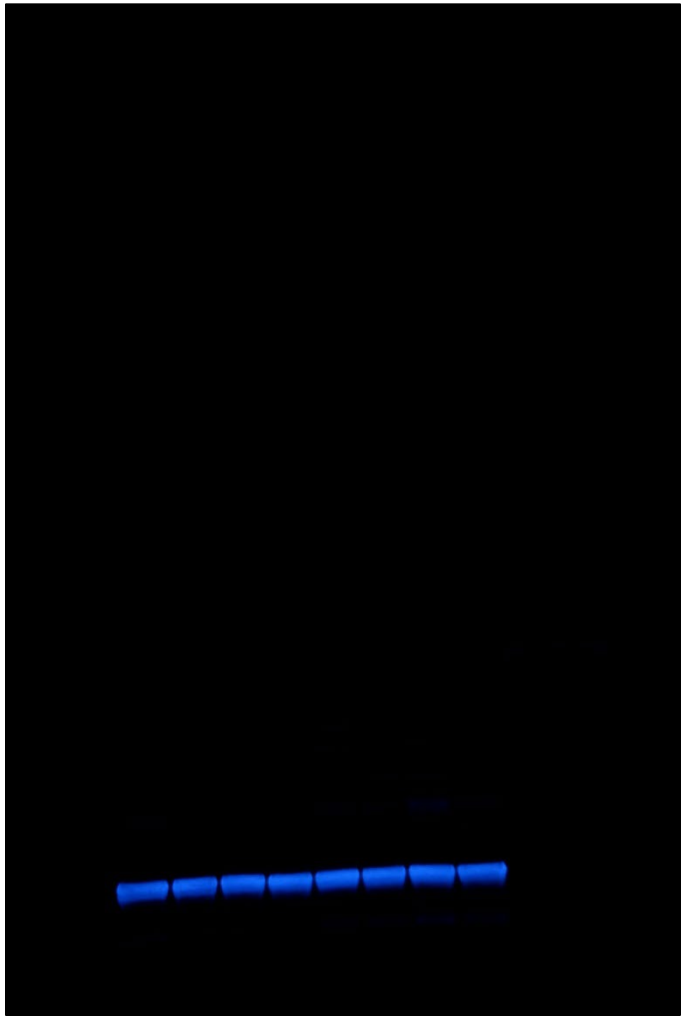

Inverted + Desaturated

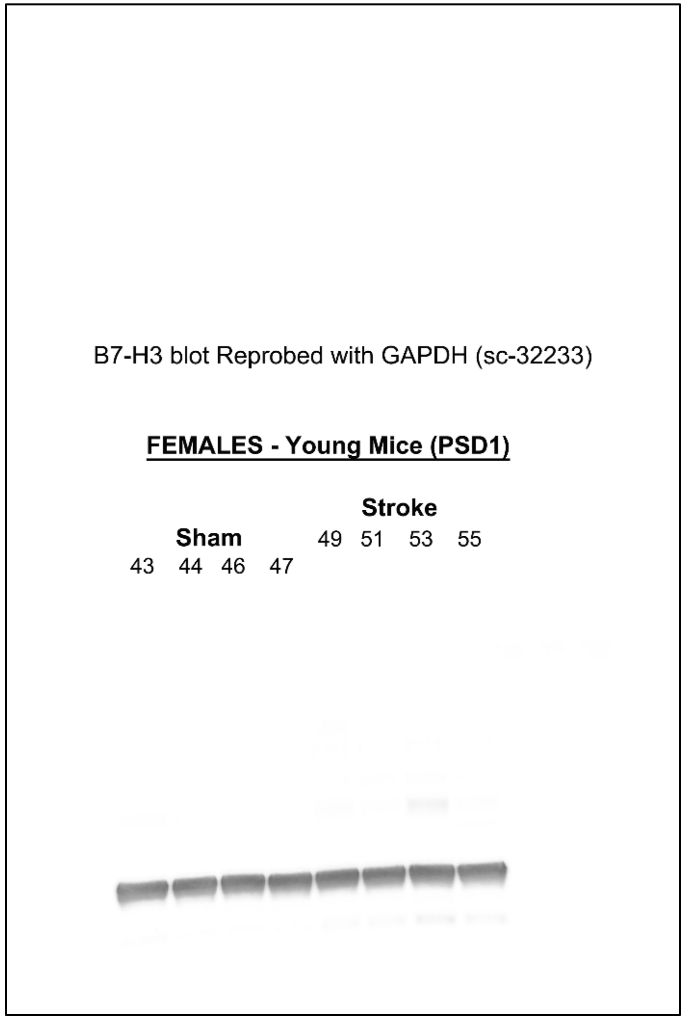

Figure 2B; Day-3

B7-H3

Bright Field Image

Chemiluminescent Image

Inverted + Desaturated

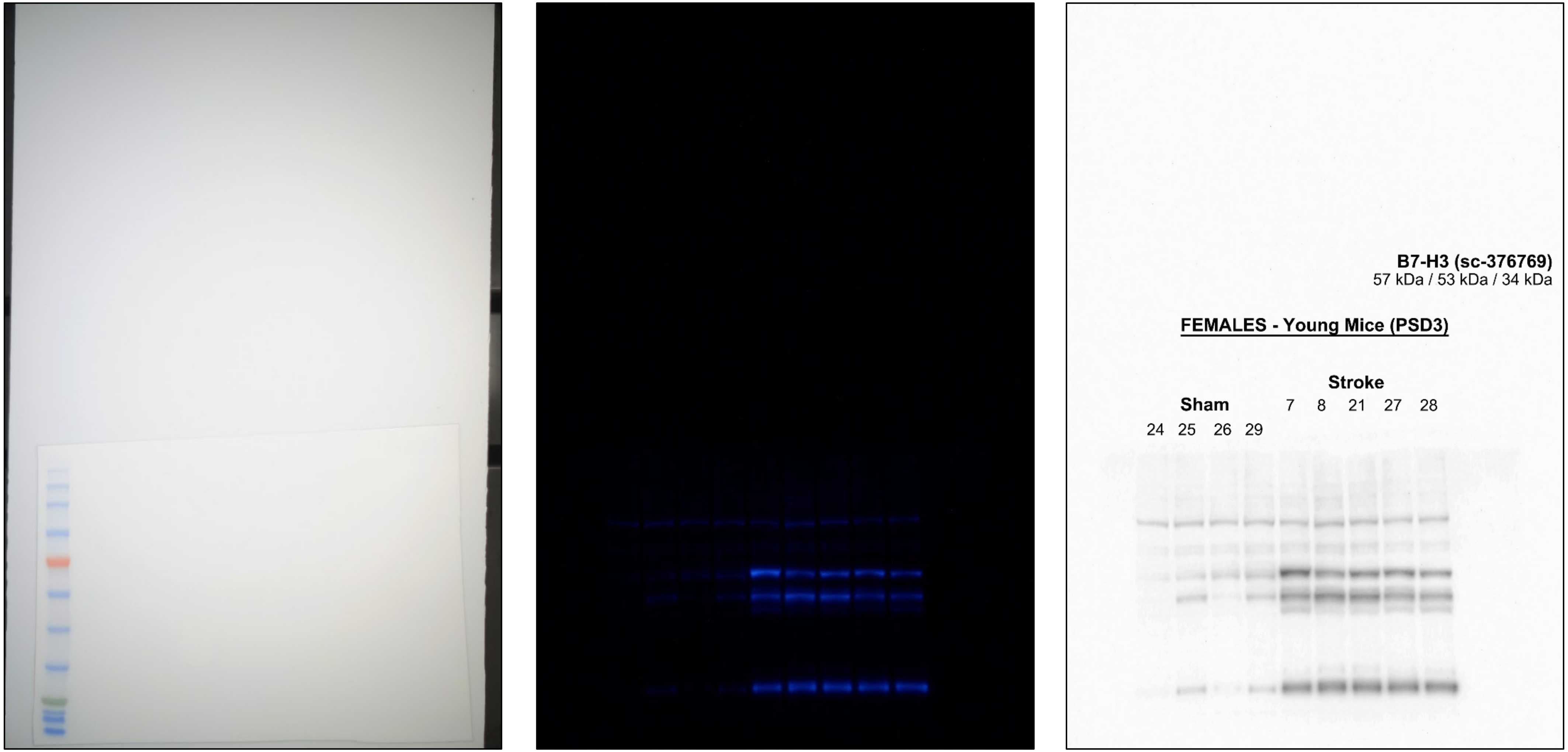

Figure 2B; Day-3

B7-H3 blot Reprobed with GAPDH

Bright Field Image

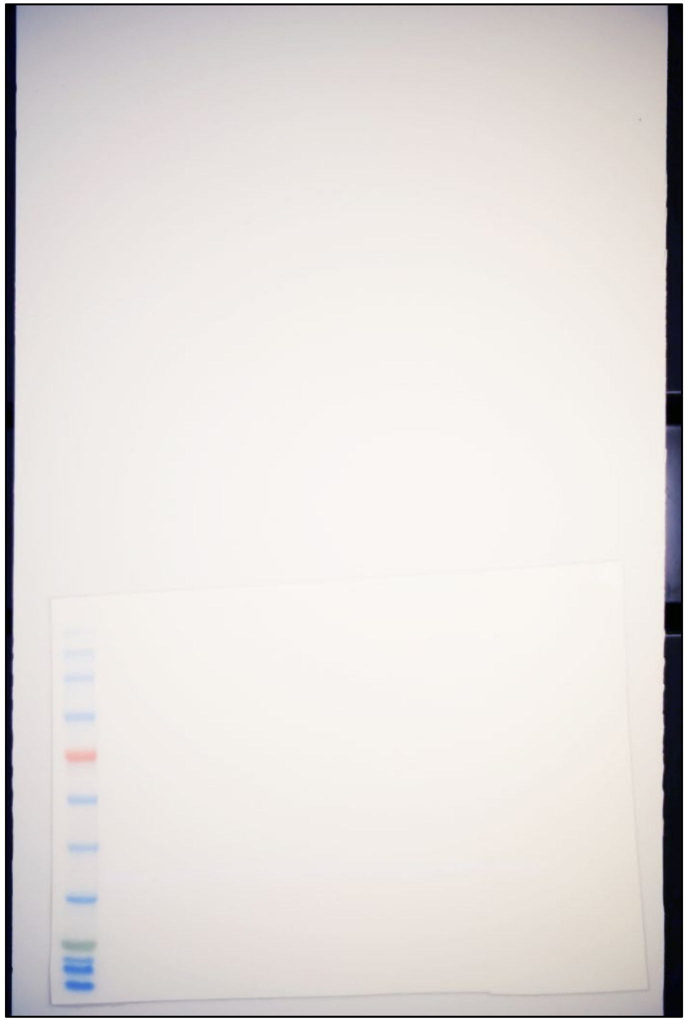

Chemiluminescent Image

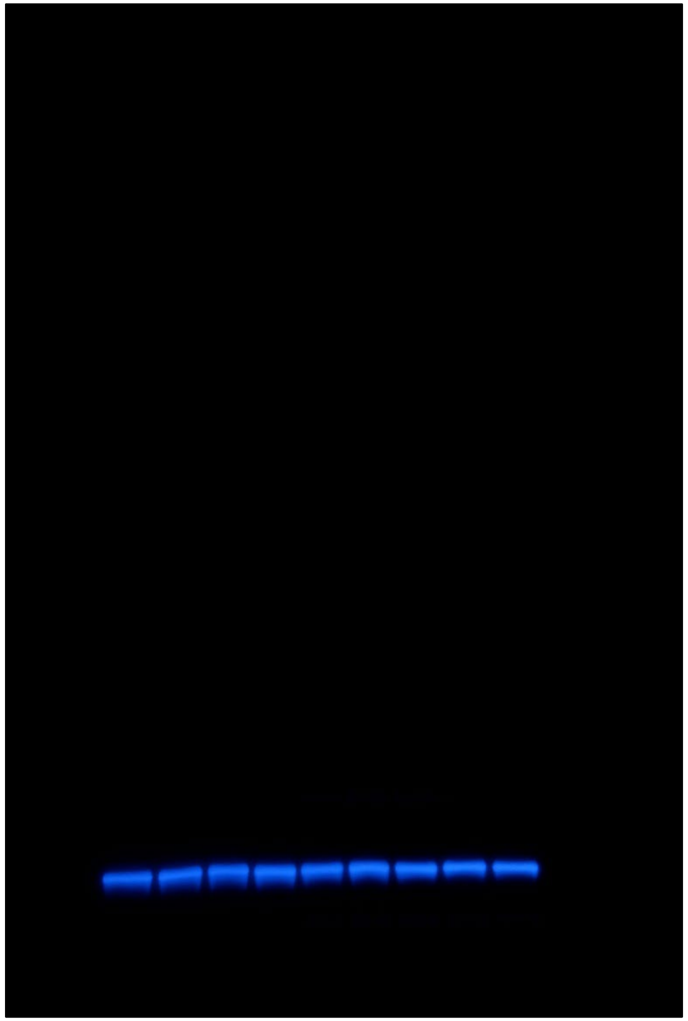

Inverted + Desaturated

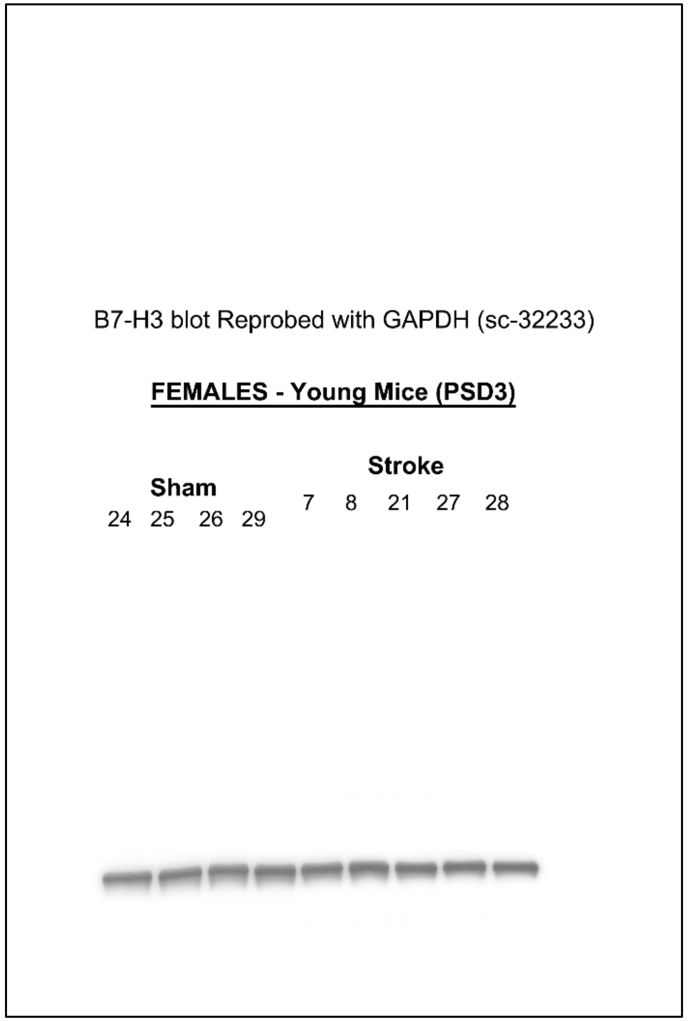

Figure 3A

B7-H3

Bright Field Image

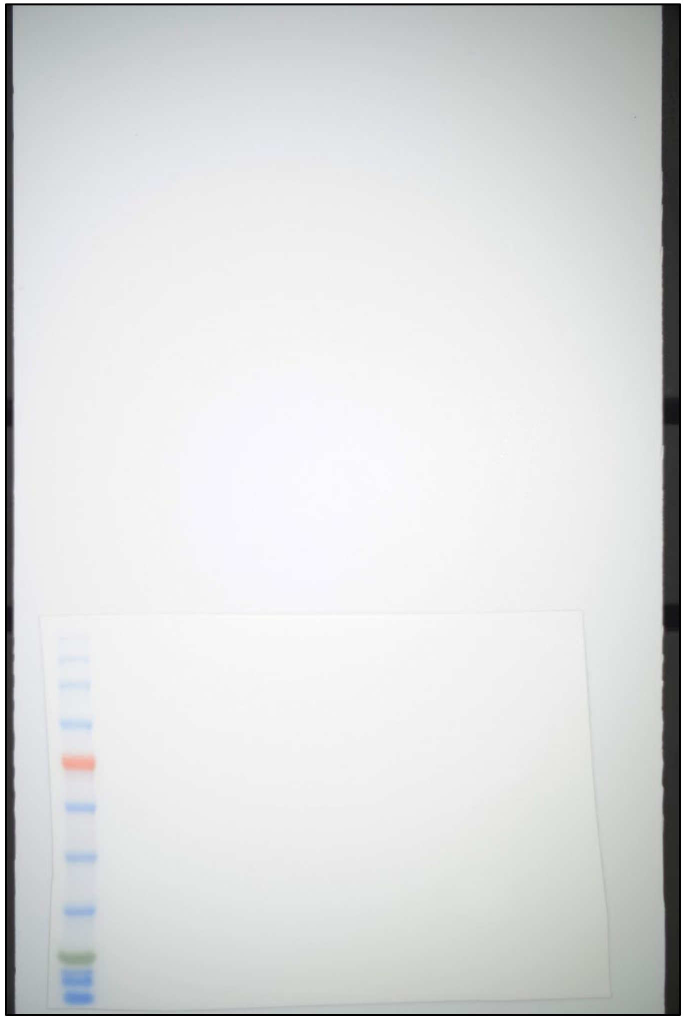

Chemiluminescent Image

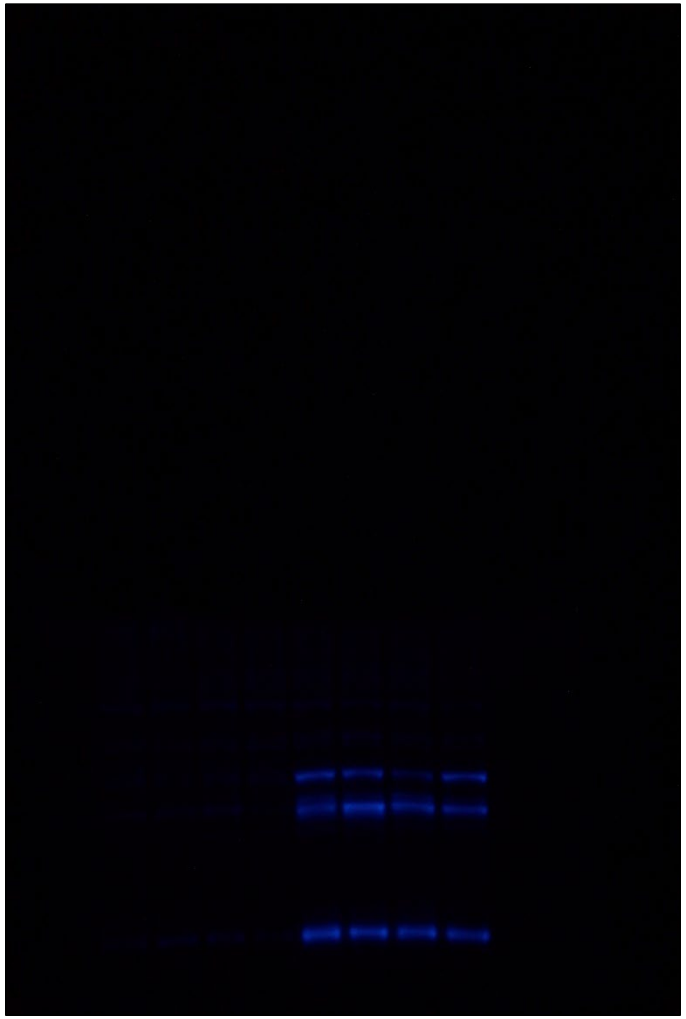

Inverted + Desaturated

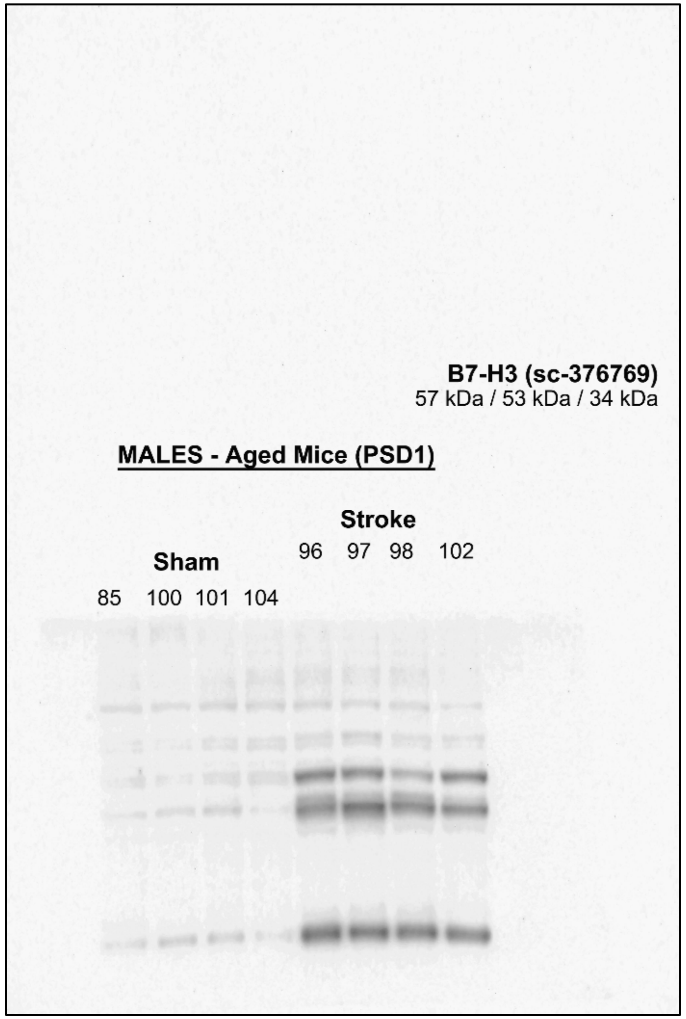

Figure 3A

B7-H3 blot Reprobed with GAPDH

Bright Field Image

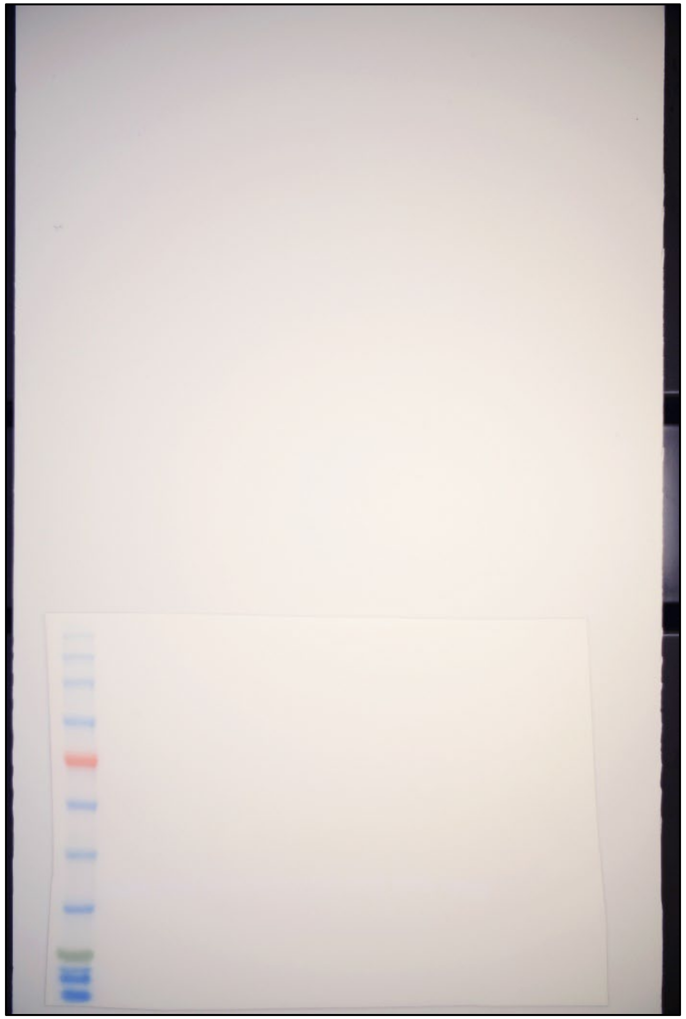

Chemiluminescent Image

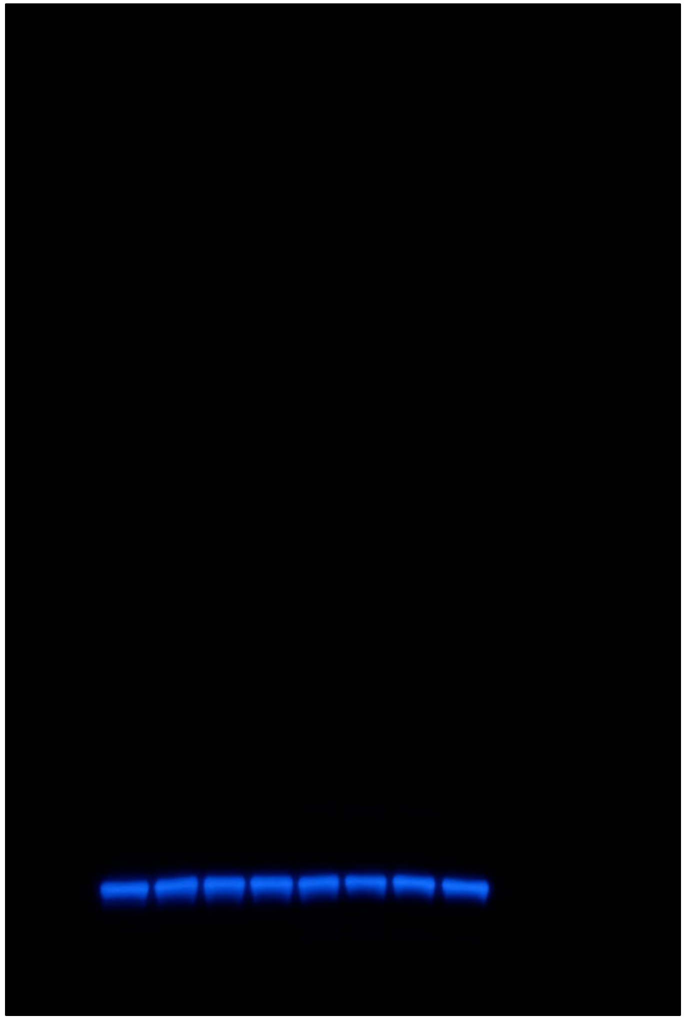

Inverted + Desaturated

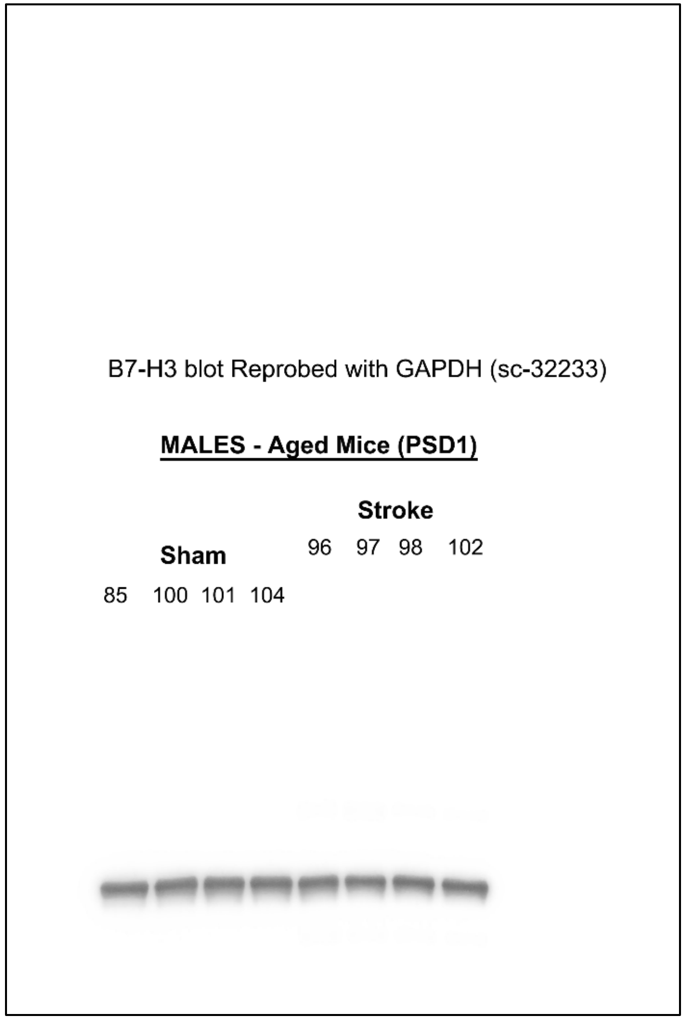

Figure 3B

B7-H3

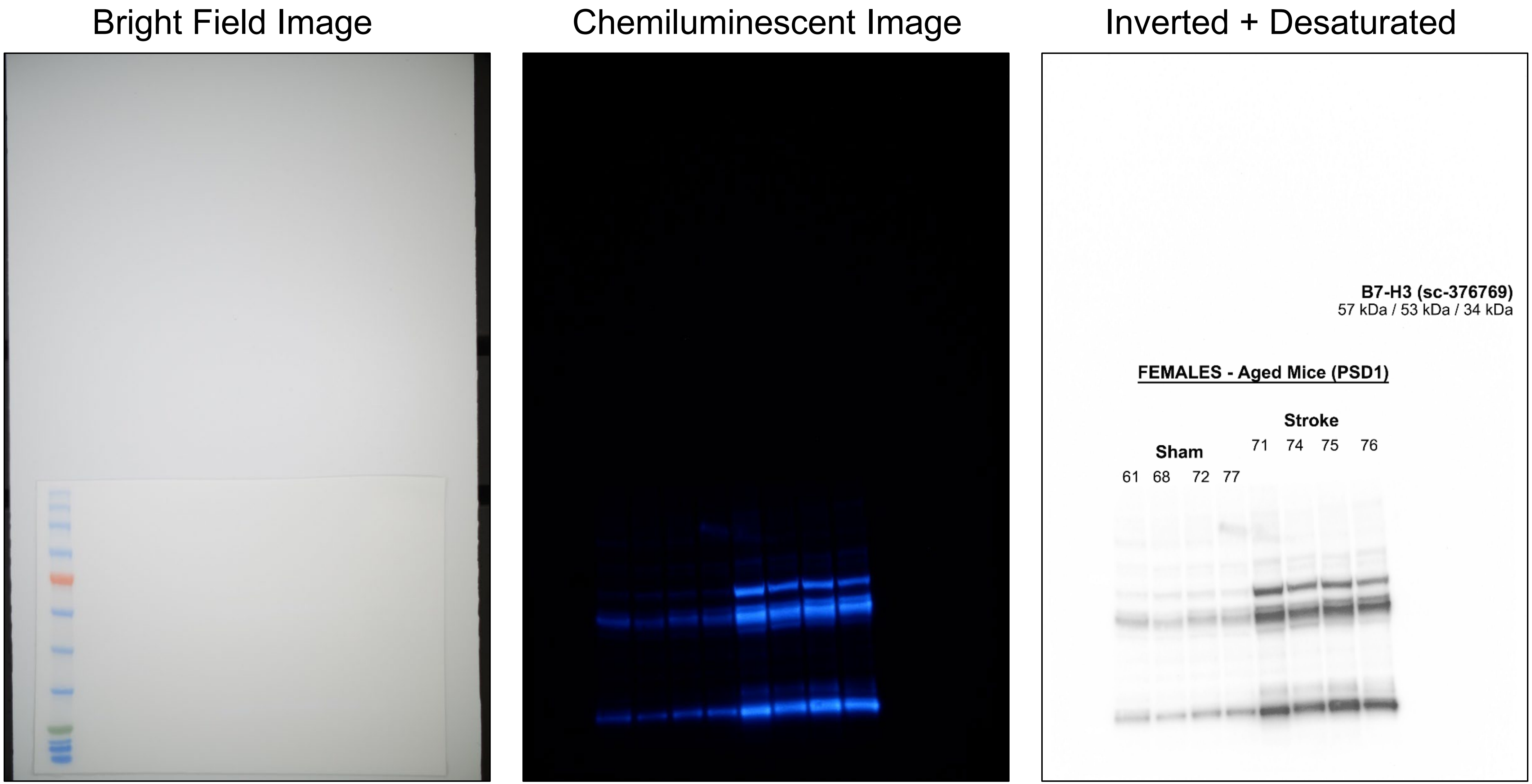

Figure 3B

B7-H3 blot Reprobed with GAPDH

Bright Field Image

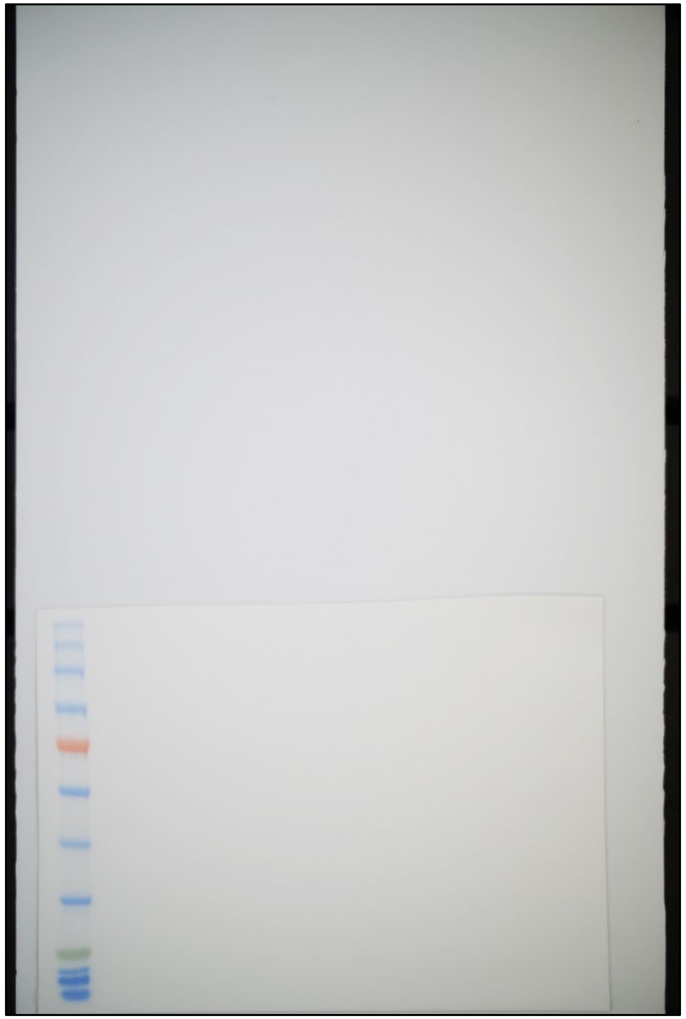

Chemiluminescent Image

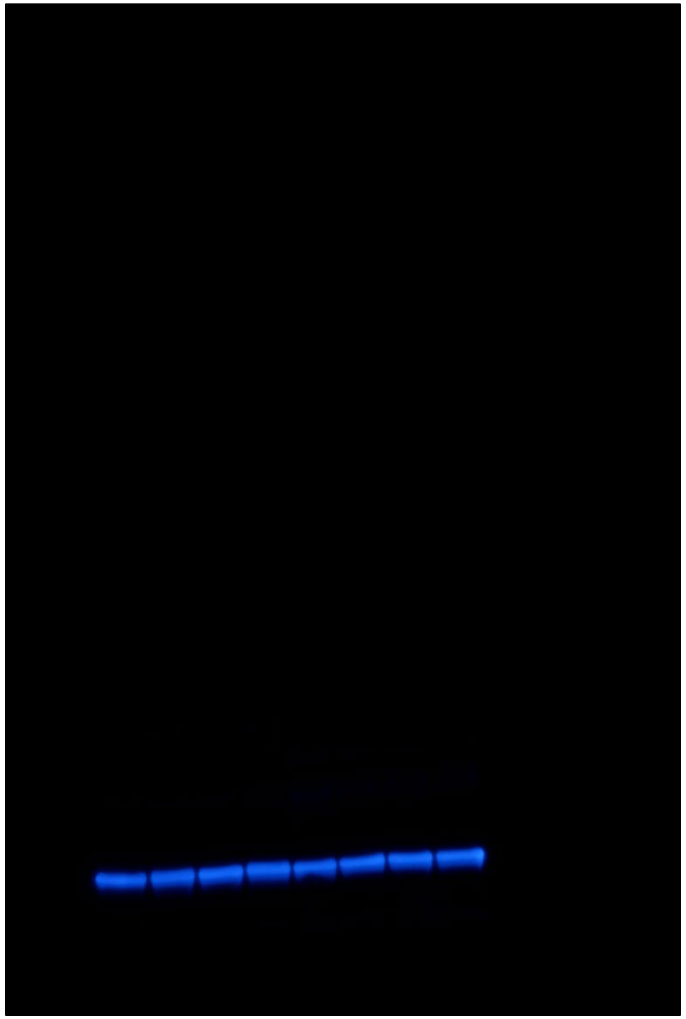

Inverted + Desaturated

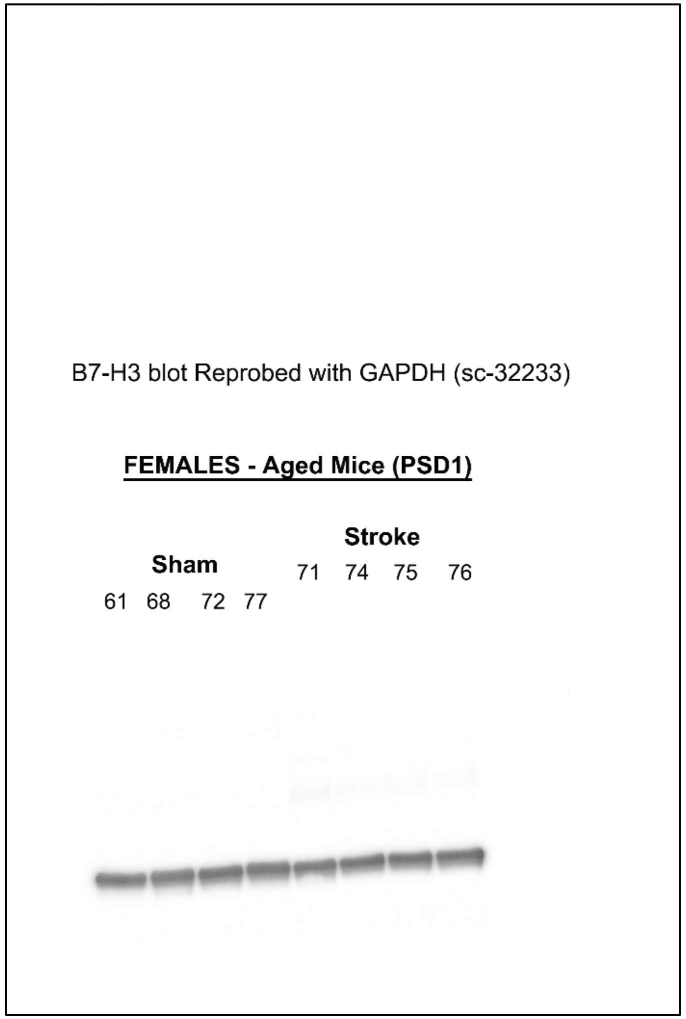

Figure 3G

B7-H3

Bright Field Image

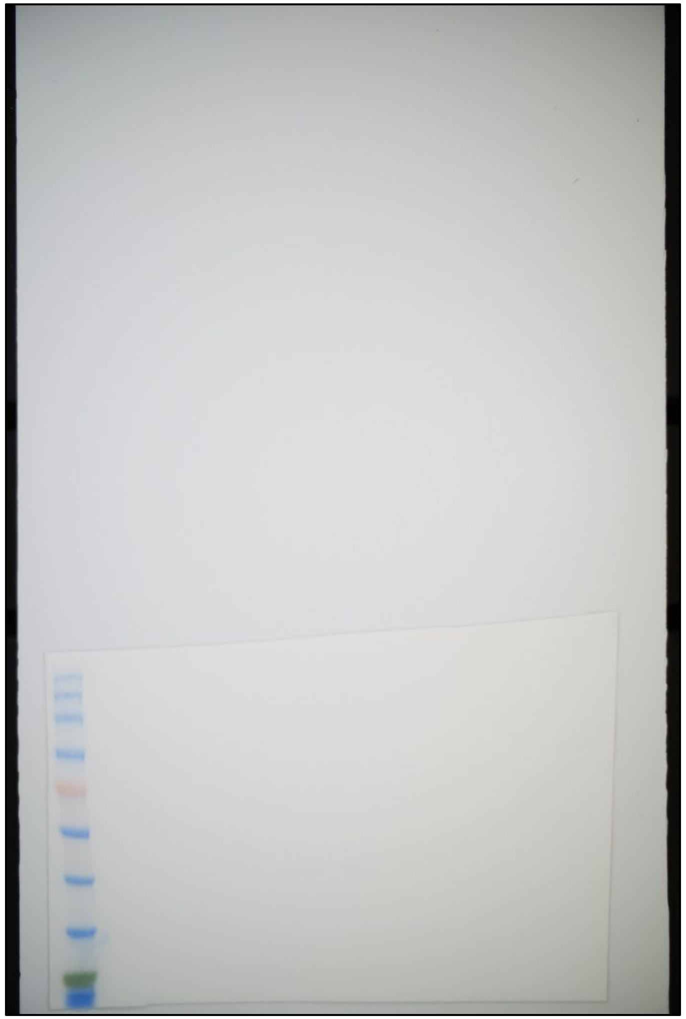

Chemiluminescent Image

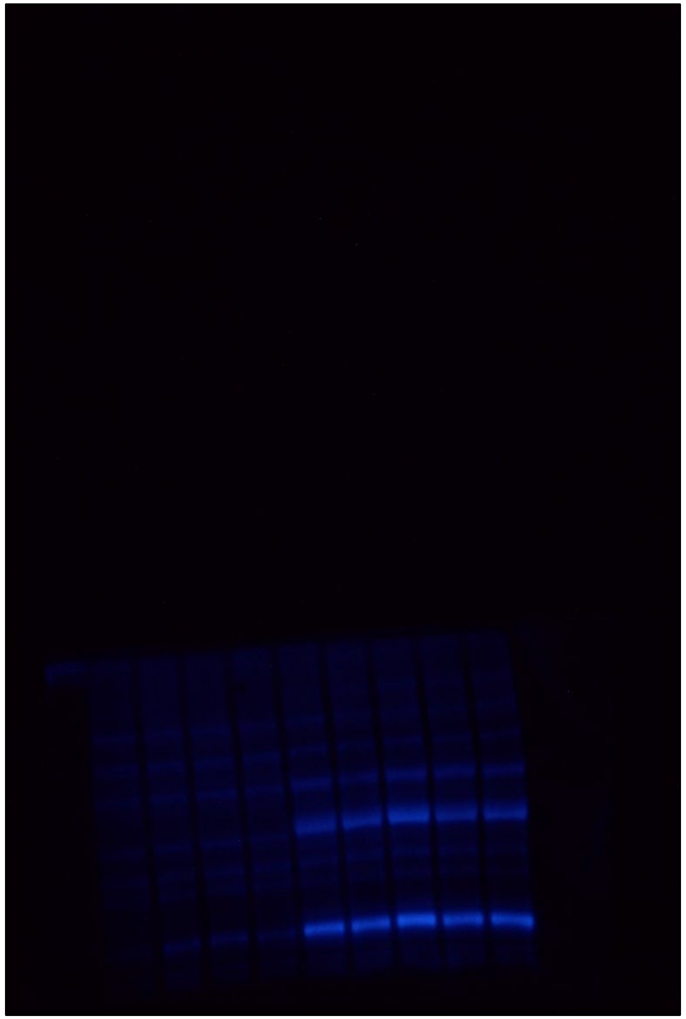

Inverted + Desaturated

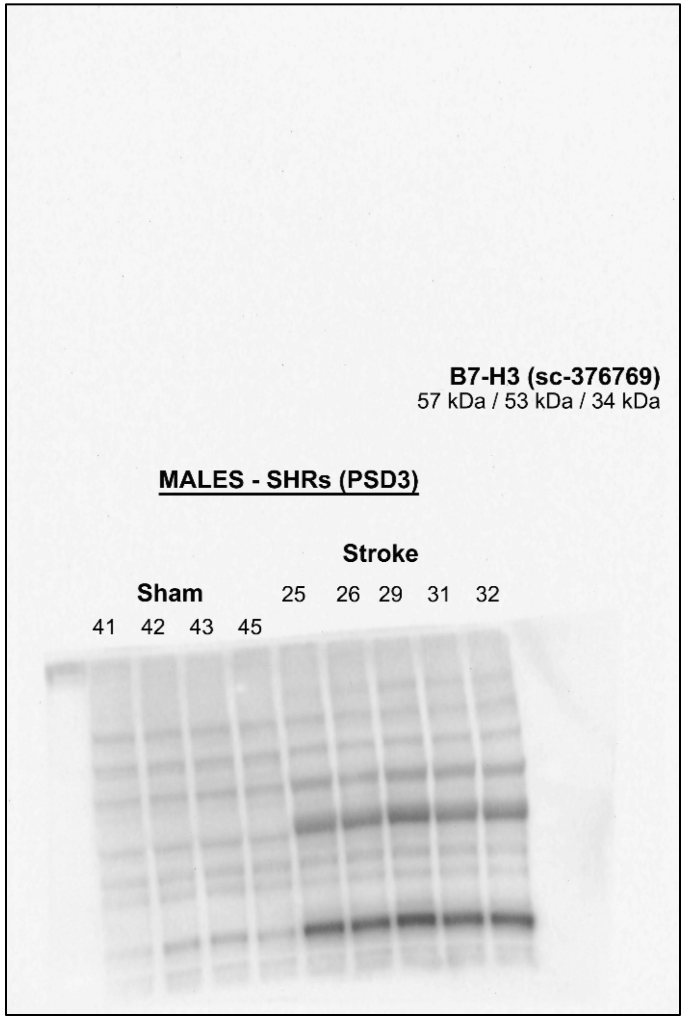

Figure 3G

B7-H3 blot Reprobed with GAPDH

Bright Field Image

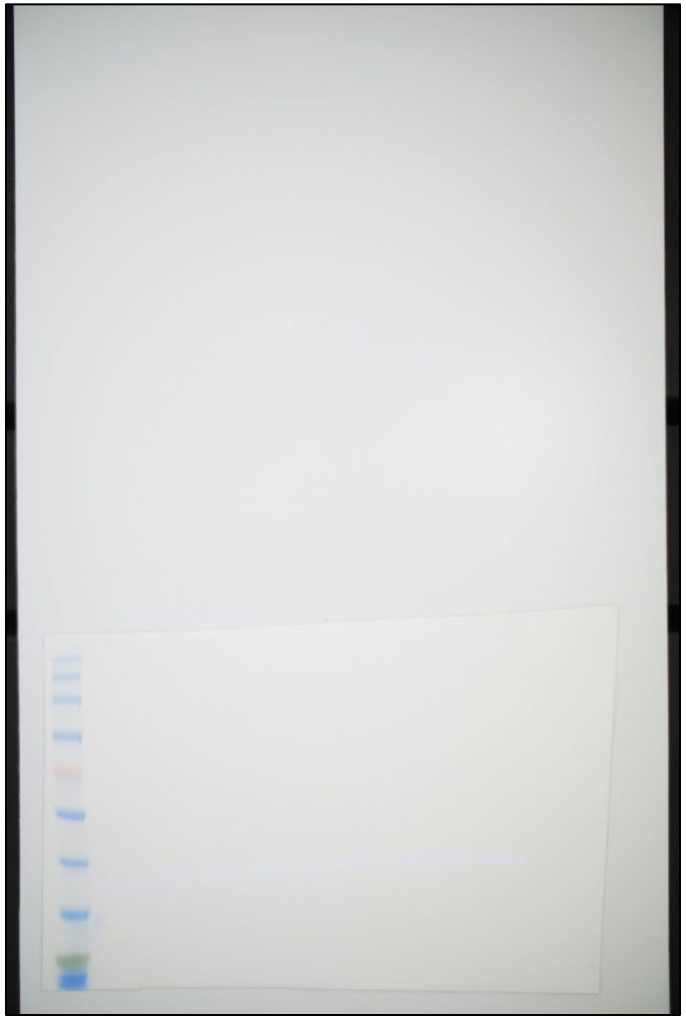

Chemiluminescent Image

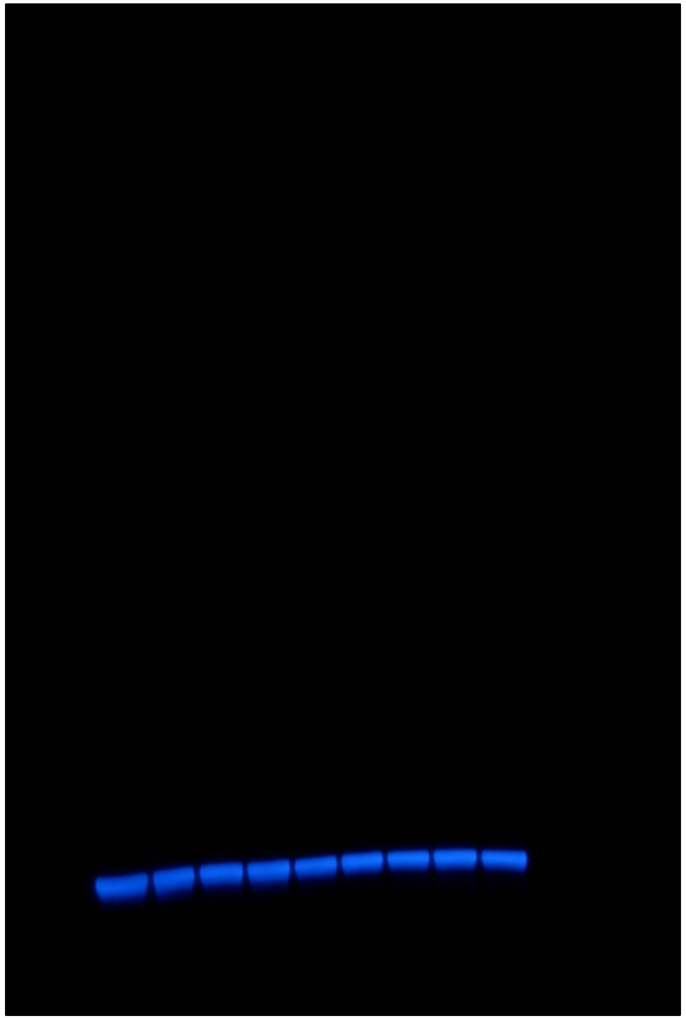

Inverted + Desaturated

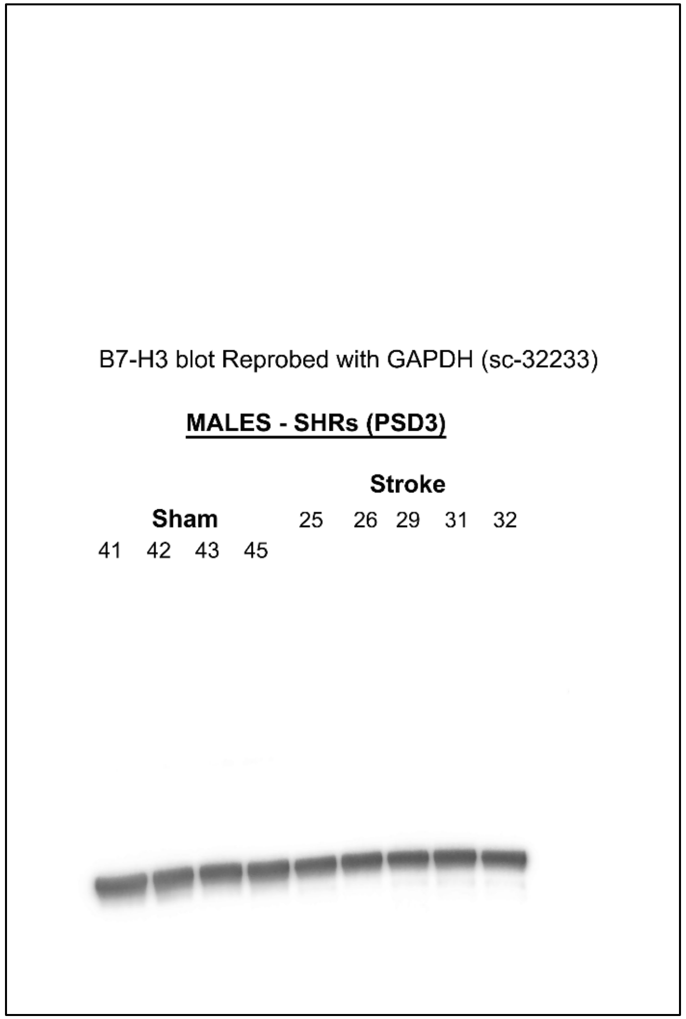

ImageJ Analysis of Western Blots

Figure 1B

Young Male Rats (Day 3)

Sham-3d

B7-H3 (57 kDa)

	MGV	IntDen
1	20.286	0.751
2	22.805	0.844
3	26.584	0.984
4	26.856	0.994
5	26.51	0.915
6		

B7-H3 (53 kDa)

	MGV	IntDen
1	17.702	0.852
2	21.568	1.038
3	28.633	1.32
4	28.512	1.314
5	25.396	1.171

B7-H3 (34 kDa)

	MGV	IntDen
1	10.313	0.422
2	14.978	0.551
3	22.642	0.88
4	17.076	0.62
5	19.892	0.671

GAPDH

	MGV	IntDen
1	67.724	2.981
2	70.923	2.589
3	67.519	2.465
4	66.862	2.274
5	76.591	2.443

B7-H3 (57 kDa)/GAPDH

	IntDen	Arbitrary
1	0.252	0.328
2	0.326	0.424
3	0.399	0.519
4	0.437	0.568
5	0.375	0.487
6	#DIV/0!	#DIV/0!

B7-H3 (53 kDa)/GAPDH

	IntDen	Arbitrary
1	0.286	0.372
2	0.401	0.521
3	0.535	0.696
4	0.578	0.751
5	0.479	0.623
6	#DIV/0!	#DIV/0!

B7-H3 (34 kDa)/GAPDH

	IntDen	Arbitrary
1	0.142	0.184
2	0.213	0.277
3	0.357	0.464
4	0.273	0.354
5	0.275	0.357
6	#DIV/0!	#DIV/0!

Stroke (2h/3d)

B7-H3 (57 kDa)

	MGV	IntDen
7	27.978	0.987
8	33.059	1.183
9	25.91	0.927
10	30.095	1.092
11	26.75	1.053
12		

B7-H3 (53 kDa)

	MGV	IntDen
7	47.247	2.495
8	53.793	2.626
9	47.225	2.305
10	60.887	2.972
11	42.174	2.173

B7-H3 (34 kDa)

	MGV	IntDen
7	58.878	2.137
8	63.913	2.32
9	52.738	1.914
10	69.077	2.507
11	57.342	2.345

GAPDH

	MGV	IntDen
7	73.531	2.46
8	69.236	2.316
9	67.37	2.306
10	63.806	2.247
11	56.656	2.189

B7-H3 (57 kDa)/GAPDH

	IntDen	Arbitrary
7	0.401	0.522
8	0.511	0.664
9	0.402	0.523
10	0.486	0.632
11	0.481	0.625
12	#DIV/0!	#DIV/0!

B7-H3 (53 kDa)/GAPDH

	IntDen	Arbitrary
7	1.014	1.318
8	1.134	1.474
9	1.000	1.299
10	1.323	1.719
11	0.993	1.290
12	#DIV/0!	#DIV/0!

B7-H3 (34 kDa)/GAPDH

	IntDen	Arbitrary
7	0.869	1.129
8	1.002	1.302
9	0.830	1.079
10	1.116	1.450
11	1.071	1.393
12	#DIV/0!	#DIV/0!

57/34
0.462
0.510
0.484
0.436
0.449
#DIV/0!

Figure 2A; Day-1

Young Male Mice (Day 1)

Sham-1d

B7-H3 (57 kDa)

	MGV	IntDen
1	20.304	0.749
2	19.222	0.709
3	16.926	0.624
4	21.216	0.782
5		
6		

B7-H3 (53 kDa)

	MGV	IntDen
1	13.88	0.787
2	12.874	0.73
3	13.135	0.744
4	16.544	0.937
5		
6		

B7-H3 (34 kDa)

	MGV	IntDen
1	15.311	0.717
2	15.955	0.747
3	14.877	0.697
4	20.67	0.968
5		
6		

GAPDH

	MGV	IntDen
1	95.676	3.619
2	91.429	3.458
3	89.043	3.368
4	94.543	3.576
5		
6		

B7-H3 (57 kDa)/GAPDH

	IntDen	Arbitrary
1	0.207	0.269
2	0.205	0.267
3	0.185	0.241
4	0.219	0.284
5	#DIV/0!	#DIV/0!
6	#DIV/0!	#DIV/0!

B7-H3 (53 kDa)/GAPDH

	IntDen	Arbitrary
1	0.217	0.283
2	0.211	0.274
3	0.221	0.287
4	0.262	0.341
5	#DIV/0!	#DIV/0!
6	#DIV/0!	#DIV/0!

B7-H3 (34 kDa)/GAPDH

	IntDen	Arbitrary
1	0.198	0.258
2	0.216	0.281
3	0.207	0.269
4	0.271	0.352
5	#DIV/0!	#DIV/0!
6	#DIV/0!	#DIV/0!

Stroke (1h/1d)

B7-H3 (57 kDa)

	MGV	IntDen
7	25.144	0.927
8	25.44	0.938
9	28.344	1.045
10	25.621	0.945
11		
12		

B7-H3 (53 kDa)

	MGV	IntDen
7	77.324	4.382
8	44.614	2.528
9	71.767	4.067
10	65.566	3.715
11		
12		

B7-H3 (34 kDa)

	MGV	IntDen
7	85.474	4.002
8	67.881	3.178
9	85.842	4.019
10	82.477	3.862
11		
12		

GAPDH

	MGV	IntDen
7	90.636	3.428
8	86.273	3.263
9	82.542	3.122
10	77.767	2.941
11		
12		

B7-H3 (57 kDa)/GAPDH

	IntDen	Arbitrary
7	0.270	0.352
8	0.287	0.374
9	0.335	0.435
10	0.321	0.418
11	#DIV/0!	#DIV/0!
12	#DIV/0!	#DIV/0!

B7-H3 (53 kDa)/GAPDH

	IntDen	Arbitrary
7	1.278	1.662
8	0.775	1.007
9	1.303	1.693
10	1.263	1.642
11	#DIV/0!	#DIV/0!
12	#DIV/0!	#DIV/0!

B7-H3 (34 kDa)/GAPDH

	IntDen	Arbitrary
7	1.167	1.518
8	0.974	1.266
9	1.287	1.674
10	1.313	1.707
11	#DIV/0!	#DIV/0!
12	#DIV/0!	#DIV/0!

57/34
0.232
0.295
0.260
0.245
#DIV/0!
#DIV/0!

Figure 2A; Day-3

Young Male Mice (Day 3)

Sham-3d

B7-H3 (57 kDa)

	MGV	IntDen
1	6.276	0.233
2	6.634	0.247
3	12.697	0.472
4	18.019	0.67
5		
6		

B7-H3 (53 kDa)

	MGV	IntDen
1	8.275	0.336
2	6.253	0.254
3	13.428	0.544
4	17.13	0.695
5		
6		

B7-H3 (34 kDa)

	MGV	IntDen
1	25.846	1.095
2	22.744	0.964
3	38.674	1.638
4	39.831	1.688
5		
6		

GAPDH

	MGV	IntDen
1	90.8	4.362
2	93.949	4.514
3	107.825	5.18
4	111.116	5.339
5		
6		

B7-H3 (57 kDa)/GAPDH

	IntDen	Arbitrary
1	0.053	0.080
2	0.055	0.082
3	0.091	0.137
4	0.125	0.188
5	#DIV/0!	#DIV/0!
6	#DIV/0!	#DIV/0!

B7-H3 (53 kDa)/GAPDH

	IntDen	Arbitrary
1	0.077	0.116
2	0.056	0.084
3	0.105	0.158
4	0.130	0.195
5	#DIV/0!	#DIV/0!
6	#DIV/0!	#DIV/0!

B7-H3 (34 kDa)/GAPDH

	IntDen	Arbitrary
1	0.251	0.377
2	0.214	0.320
3	0.316	0.474
4	0.316	0.474
5	#DIV/0!	#DIV/0!
6	#DIV/0!	#DIV/0!

Stroke (1h/3d)

B7-H3 (57 kDa)

	MGV	IntDen
7	130.065	4.838
8	108.753	4.046
9	113.469	4.221
10	109.263	4.065
11		
12		

B7-H3 (53 kDa)

	MGV	IntDen
7	55.148	2.236
8	73.81	2.993
9	81.5	3.304
10	94.661	3.838
11		
12		

B7-H3 (34 kDa)

	MGV	IntDen
7	109.533	4.641
8	122.033	5.17
9	119.666	5.07
10	150.117	6.36
11		
12		

GAPDH

	MGV	IntDen
7	101.495	4.876
8	108.852	5.23
9	112.371	5.399
10	115.094	5.53
11		
12		

B7-H3 (57 kDa)/GAPDH

	IntDen	Arbitrary
7	0.992	1.488
8	0.774	1.160
9	0.782	1.173
10	0.735	1.103
11	#DIV/0!	#DIV/0!
12	#DIV/0!	#DIV/0!

B7-H3 (53 kDa)/GAPDH

	IntDen	Arbitrary
7	0.459	0.688
8	0.572	0.858
9	0.612	0.918
10	0.694	1.041
11	#DIV/0!	#DIV/0!
12	#DIV/0!	#DIV/0!

B7-H3 (34 kDa)/GAPDH

	IntDen	Arbitrary
7	0.952	1.428
8	0.989	1.483
9	0.939	1.409
10	1.150	1.725
11	#DIV/0!	#DIV/0!
12	#DIV/0!	#DIV/0!

57/34
1.042
0.783
0.833
0.639
#DIV/0!
#DIV/0!

Figure 2B; Day-1

Young Female Mice (Day 1)

Sham-1d

B7-H3 (57 kDa)			B7-H3 (53 kDa)			B7-H3 (34 kDa)			GAPDH		
	MGV	IntDen		MGV	IntDen		MGV	IntDen		MGV	IntDen
1	34.137	1.797	1	47.666	3.563	1	54.053	2.976	1	56.85	2.972
2	30.209	1.503	2	32.004	2.044	2	43.015	1.708	2	62.959	2.681
3	37.431	1.819	3	33.141	2.068	3	37.096	1.473	3	68.66	2.823
4	35.168	1.807	4	24.56	1.514	4	32.268	1.281	4	68.594	2.82

B7-H3 (57 kDa)/GAPDH			B7-H3 (53 kDa)/GAPDH			B7-H3 (34 kDa)/GAPDH		
	IntDen	Arbitrary		IntDen	Arbitrary		IntDen	Arbitrary
1	0.605	0.484	1	1.199	0.959	1	1.001	0.801
2	0.561	0.448	2	0.762	0.610	2	0.637	0.510
3	0.644	0.515	3	0.733	0.586	3	0.522	0.417
4	0.641	0.513	4	0.537	0.430	4	0.454	0.363
5	#DIV/0!	#DIV/0!	5	#DIV/0!	#DIV/0!	5	#DIV/0!	#DIV/0!
6	#DIV/0!	#DIV/0!	6	#DIV/0!	#DIV/0!	6	#DIV/0!	#DIV/0!

Stroke (1h/1d)

B7-H3 (57 kDa)			B7-H3 (53 kDa)			B7-H3 (34 kDa)			GAPDH		
	MGV	IntDen		MGV	IntDen		MGV	IntDen		MGV	IntDen
7	70.094	4.257	7	63.14	3.893	7	73.689	3.696	7	74.25	2.879
8	86.999	5.622	8	47.797	3.037	8	73.055	3.665	8	72.197	2.766
9	93.296	6.392	9	93.277	7.121	9	101.198	5.324	9	70.995	3.023
10	67.348	4.614	10	59.273	4.185	10	86.131	4.795	10	63.477	2.92

B7-H3 (57 kDa)/GAPDH			B7-H3 (53 kDa)/GAPDH			B7-H3 (34 kDa)/GAPDH		
	IntDen	Arbitrary		IntDen	Arbitrary		IntDen	Arbitrary
7	1.479	1.183	7	1.352	1.082	7	1.284	1.027
8	2.033	1.626	8	1.098	0.878	8	1.325	1.060
9	2.114	1.692	9	2.356	1.884	9	1.761	1.409
10	1.580	1.264	10	1.433	1.147	10	1.642	1.314
11	#DIV/0!	#DIV/0!	11	#DIV/0!	#DIV/0!	11	#DIV/0!	#DIV/0!
12	#DIV/0!	#DIV/0!	12	#DIV/0!	#DIV/0!	12	#DIV/0!	#DIV/0!

57/34
1.152
1.534
1.201
0.962
#DIV/0!
#DIV/0!

Figure 2B; Day-3

Young Female Mice (Day 3)

Sham-3d

B7-H3 (57 kDa)

	MGV	IntDen
1	18.219	0.638
2	30.368	1.063
3	37.841	1.324
4	48.846	1.71
5		
6		

B7-H3 (53 kDa)

	MGV	IntDen
1	20.017	0.616
2	54.947	1.691
3	29.854	0.919
4	58.22	1.791
5		
6		

B7-H3 (34 kDa)

	MGV	IntDen
1	16.819	0.484
2	36.407	1.048
3	20.693	0.595
4	36.583	1.053
5		
6		

GAPDH

	MGV	IntDen
1	81.632	2.571
2	83.418	2.628
3	82.798	2.608
4	88.309	2.782
5		
6		

B7-H3 (57 kDa)/GAPDH

	IntDen	Arbitrary
1	0.248	0.298
2	0.404	0.485
3	0.508	0.609
4	0.615	0.738
5	#DIV/0!	#DIV/0!
6	#DIV/0!	#DIV/0!

B7-H3 (53 kDa)/GAPDH

	IntDen	Arbitrary
1	0.240	0.288
2	0.643	0.772
3	0.352	0.423
4	0.644	0.773
5	#DIV/0!	#DIV/0!
6	#DIV/0!	#DIV/0!

B7-H3 (34 kDa)/GAPDH

	IntDen	Arbitrary
1	0.188	0.226
2	0.399	0.479
3	0.228	0.274
4	0.379	0.454
5	#DIV/0!	#DIV/0!
6	#DIV/0!	#DIV/0!

Stroke (1h/3d)

B7-H3 (57 kDa)

	MGV	IntDen
7	107.892	3.776
8	79.89	2.796
9	85.137	2.98
10	80.532	2.819
11	67.9	2.377
12		

B7-H3 (53 kDa)

	MGV	IntDen
7	103.113	3.172
8	116.007	3.569
9	113.112	3.48
10	94.46	2.906
11	89.824	2.764
12		

B7-H3 (34 kDa)

	MGV	IntDen
7	89.034	2.562
8	97.065	2.793
9	98.495	2.834
10	96.024	2.763
11	95.79	2.757
12		

GAPDH

	MGV	IntDen
7	82.945	2.613
8	83.38	2.626
9	81.463	2.566
10	80.296	2.529
11	78.491	2.472
12		

B7-H3 (57 kDa)/GAPDH

	IntDen	Arbitrary
7	1.445	1.734
8	1.065	1.278
9	1.161	1.394
10	1.115	1.338
11	0.962	1.154
12	#DIV/0!	#DIV/0!

B7-H3 (53 kDa)/GAPDH

	IntDen	Arbitrary
7	1.214	1.457
8	1.359	1.631
9	1.356	1.627
10	1.149	1.379
11	1.118	1.342
12	#DIV/0!	#DIV/0!

B7-H3 (34 kDa)/GAPDH

	IntDen	Arbitrary
7	0.980	1.177
8	1.064	1.276
9	1.104	1.325
10	1.093	1.311
11	1.115	1.338
12	#DIV/0!	#DIV/0!

57/34
1.474
1.001
1.052
1.020
0.862
#DIV/0!

Figure 3C

Aged Male Mice (Day 1)

Sham-1d

B7-H3 (57 kDa)			B7-H3 (53 kDa)			B7-H3 (34 kDa)			GAPDH		
	MGV	IntDen		MGV	IntDen		MGV	IntDen		MGV	IntDen
1	21.291	1.261	1	23.905	1.371	1	20.084	0.921	1	72.153	2.893
2	19.509	1.156	2	27.674	1.587	2	25.223	1.157	2	72.083	2.89
3	24.672	1.462	3	31.637	1.814	3	24.901	1.142	3	72.18	2.894
4	27.43	1.625	4	26.291	1.507	4	20.791	0.954	4	72.396	2.902
5			5			5			5		
6			6			6			6		

B7-H3 (57 kDa)/GAPDH			B7-H3 (53 kDa)/GAPDH			B7-H3 (34 kDa)/GAPDH		
	IntDen	Arbitrary		IntDen	Arbitrary		IntDen	Arbitrary
1	0.436	0.349	1	0.474	0.379	1	0.318	0.255
2	0.400	0.320	2	0.549	0.439	2	0.400	0.320
3	0.505	0.404	3	0.627	0.501	3	0.395	0.316
4	0.560	0.448	4	0.519	0.415	4	0.329	0.263
5	#DIV/0!	#DIV/0!	5	#DIV/0!	#DIV/0!	5	#DIV/0!	#DIV/0!
6	#DIV/0!	#DIV/0!	6	#DIV/0!	#DIV/0!	6	#DIV/0!	#DIV/0!

Stroke (1h/1d)

B7-H3 (57 kDa)			B7-H3 (53 kDa)			B7-H3 (34 kDa)			GAPDH		
	MGV	IntDen		MGV	IntDen		MGV	IntDen		MGV	IntDen
7	73.511	4.355	7	83.513	4.788	7	85.533	3.923	7	68.838	2.76
8	68.372	4.051	8	96.483	5.532	8	81.892	3.756	8	63.634	2.551
9	47.497	2.814	9	83.836	4.807	9	78.756	3.612	9	69.489	2.786
10	68.408	4.053	10	71.14	4.079	10	71.471	3.278	10	72.993	2.926
11			11			11			11		
12			12			12			12		

B7-H3 (57 kDa)/GAPDH			B7-H3 (53 kDa)/GAPDH			B7-H3 (34 kDa)/GAPDH		
	IntDen	Arbitrary		IntDen	Arbitrary		IntDen	Arbitrary
7	1.578	1.262	7	1.735	1.388	7	1.421	1.137
8	1.588	1.270	8	2.169	1.735	8	1.472	1.178
9	1.010	0.808	9	1.725	1.380	9	1.296	1.037
10	1.385	1.108	10	1.394	1.115	10	1.120	0.896
11	#DIV/0!	#DIV/0!	11	#DIV/0!	#DIV/0!	11	#DIV/0!	#DIV/0!
12	#DIV/0!	#DIV/0!	12	#DIV/0!	#DIV/0!	12	#DIV/0!	#DIV/0!

57/34
1.110
1.079
0.779
1.236
#DIV/0!
#DIV/0!

Figure 3D

Aged Female Mice (Day 1)

Sham-1d

B7-H3 (57 kDa)

	MGV	IntDen
1	8.318	0.345
2	10.704	0.444
3	12.293	0.51
4	12.807	0.531
5		
6		

B7-H3 (53 kDa)

	MGV	IntDen
1	34.746	1.524
2	26.36	1.156
3	37.169	1.63
4	43.238	1.897
5		
6		

B7-H3 (34 kDa)

	MGV	IntDen
1	31.649	1.269
2	25.62	1.027
3	38.589	1.547
4	50.641	2.03
5		
6		

GAPDH

	MGV	IntDen
1	86.598	3.799
2	86.237	3.783
3	91.106	3.997
4	92.309	4.049
5		
6		

B7-H3 (57 kDa)/GAPDH

	IntDen	Arbitrary
1	0.091	0.118
2	0.117	0.153
3	0.128	0.166
4	0.131	0.170
5	#DIV/0!	#DIV/0!
6	#DIV/0!	#DIV/0!

B7-H3 (53 kDa)/GAPDH

	IntDen	Arbitrary
1	0.401	0.522
2	0.306	0.397
3	0.408	0.530
4	0.469	0.609
5	#DIV/0!	#DIV/0!
6	#DIV/0!	#DIV/0!

B7-H3 (34 kDa)/GAPDH

	IntDen	Arbitrary
1	0.334	0.434
2	0.271	0.353
3	0.387	0.503
4	0.501	0.652
5	#DIV/0!	#DIV/0!
6	#DIV/0!	#DIV/0!

Stroke (1h/1d)

B7-H3 (57 kDa)

	MGV	IntDen
7	96.925	4.022
8	73.021	3.03
9	81.62	3.387
10	63.938	2.653
11		
12		

B7-H3 (53 kDa)

	MGV	IntDen
7	118.847	5.213
8	107.685	4.724
9	117.618	5.16
10	112.891	4.952
11		
12		

B7-H3 (34 kDa)

	MGV	IntDen
7	110.415	4.426
8	101.171	4.056
9	122.674	4.918
10	110.586	4.433
11		
12		

GAPDH

	MGV	IntDen
7	83.048	3.643
8	88.361	3.876
9	89.233	3.914
10	93.651	4.108
11		
12		

B7-H3 (57 kDa)/GAPDH

	IntDen	Arbitrary
7	1.104	1.435
8	0.782	1.016
9	0.865	1.125
10	0.646	0.840
11	#DIV/0!	#DIV/0!
12	#DIV/0!	#DIV/0!

B7-H3 (53 kDa)/GAPDH

	IntDen	Arbitrary
7	1.431	1.860
8	1.219	1.584
9	1.318	1.714
10	1.205	1.567
11	#DIV/0!	#DIV/0!
12	#DIV/0!	#DIV/0!

B7-H3 (34 kDa)/GAPDH

	IntDen	Arbitrary
7	1.215	1.579
8	1.046	1.360
9	1.257	1.633
10	1.079	1.403
11	#DIV/0!	#DIV/0!
12	#DIV/0!	#DIV/0!

57/34
0.909
0.747
0.689
0.598
#DIV/0!
#DIV/0!

Figure 3H

Male SHRs (Day 3)

Sham-3d

B7-H3 (57 kDa)

	MGV	IntDen
1	31.33	1.099
2	38.393	1.347
3	43.577	1.529
4	42.527	1.492
5		
6		

B7-H3 (53 kDa)

	MGV	IntDen
1	25.108	0.904
2	33.654	1.211
3	39.039	1.405
4	39.406	1.418
5		

B7-H3 (34 kDa)

	MGV	IntDen
1	20.125	0.697
2	33.308	1.153
3	37.253	1.29
4	38.771	1.342
5		

GAPDH

	MGV	IntDen
1	115.834	4.402
2	107.95	4.102
3	110.348	4.193
4	110.803	4.211
5		

B7-H3 (57 kDa)/GAPDH

	IntDen	Arbitrary
1	0.250	0.424
2	0.328	0.558
3	0.365	0.620
4	0.354	0.602
5	#DIV/0!	#DIV/0!
6	#DIV/0!	#DIV/0!

B7-H3 (53 kDa)/GAPDH

	IntDen	Arbitrary
1	0.205	0.349
2	0.295	0.502
3	0.335	0.570
4	0.337	0.572
5	#DIV/0!	#DIV/0!
6	#DIV/0!	#DIV/0!

B7-H3 (34 kDa)/GAPDH

	IntDen	Arbitrary
1	0.158	0.269
2	0.281	0.478
3	0.308	0.523
4	0.319	0.542
5	#DIV/0!	#DIV/0!
6	#DIV/0!	#DIV/0!

Stroke (1h/3d)

B7-H3 (57 kDa)

	MGV	IntDen
7	56.034	1.966
8	49.561	1.738
9	60.732	2.13
10	54.317	1.905
11	48.568	1.704
12		

B7-H3 (53 kDa)

	MGV	IntDen
7	85.952	3.093
8	88.122	3.171
9	103.633	3.73
10	83.301	2.998
11	82.878	2.983

B7-H3 (34 kDa)

	MGV	IntDen
7	101.889	3.528
8	103.396	3.58
9	117.14	4.056
10	103.426	3.581
11	102.426	3.546

GAPDH

	MGV	IntDen
7	104.866	3.985
8	107.813	4.097
9	106.581	4.05
10	110.33	4.193
11	103.997	3.952

B7-H3 (57 kDa)/GAPDH

	IntDen	Arbitrary
7	0.493	0.839
8	0.424	0.721
9	0.526	0.894
10	0.454	0.772
11	0.431	0.733
12	#DIV/0!	#DIV/0!

B7-H3 (53 kDa)/GAPDH

	IntDen	Arbitrary
7	0.776	1.319
8	0.774	1.316
9	0.921	1.566
10	0.715	1.216
11	0.755	1.283
12	#DIV/0!	#DIV/0!

B7-H3 (34 kDa)/GAPDH

	IntDen	Arbitrary
7	0.885	1.505
8	0.874	1.485
9	1.001	1.703
10	0.854	1.452
11	0.897	1.525
12	#DIV/0!	#DIV/0!

57/34
0.557
0.485
0.525
0.532
0.481
#DIV/0!

Brain Sections Processed by IHC

B7-H3 - DAB Staining

Young Male Rats – <u>SHAM Group</u>

Figure 5A

SDRM-369

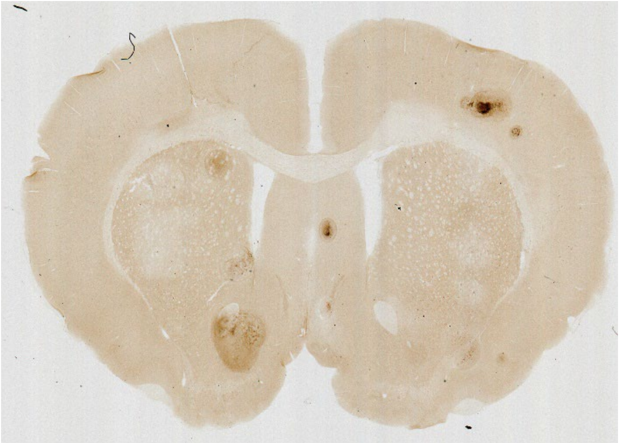

SDRM-375

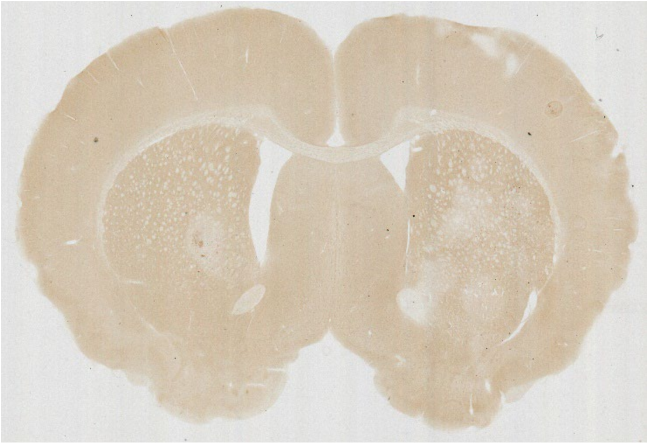

SDRM-376

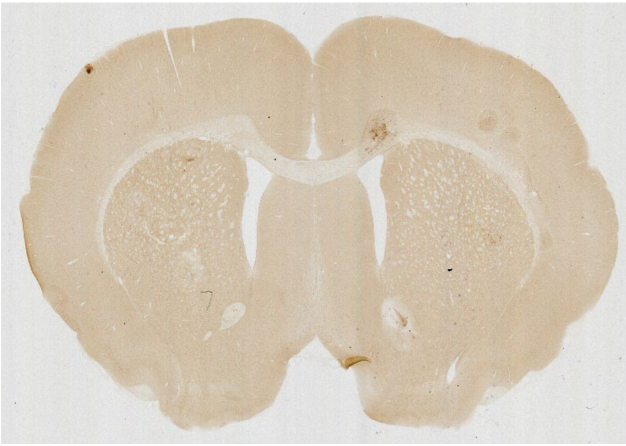

SDRM-384

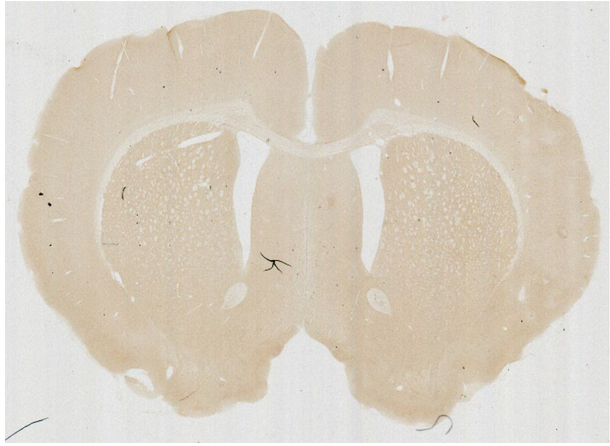

SDRM-394

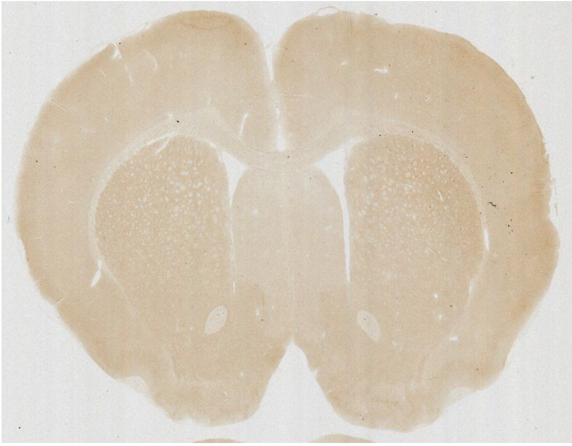

SDRM-467

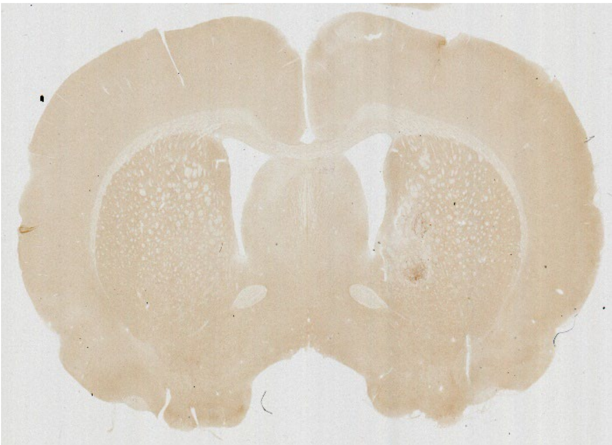

Figure 5A

Young Male Rats – <u>STROKE Group</u>

SDRM-372

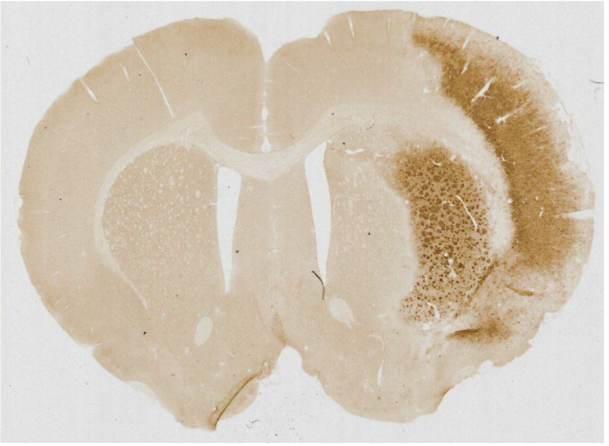

SDRM-380

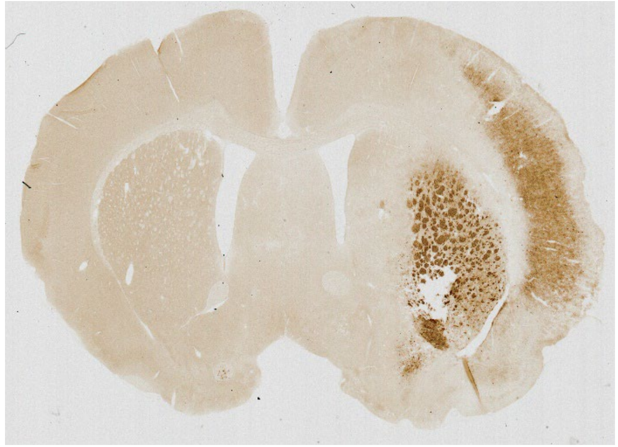

SDRM-382

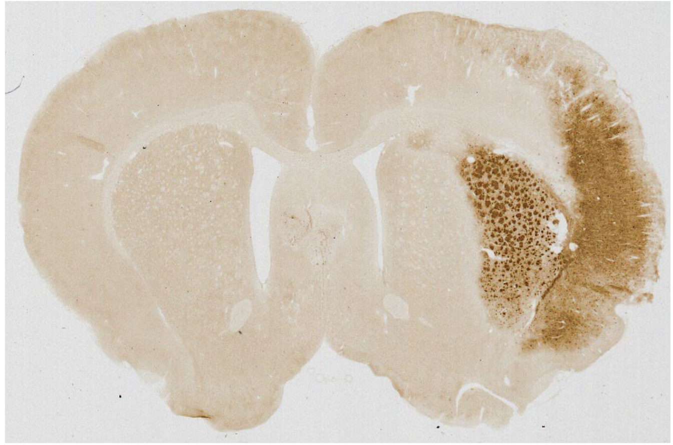

SDRM-415

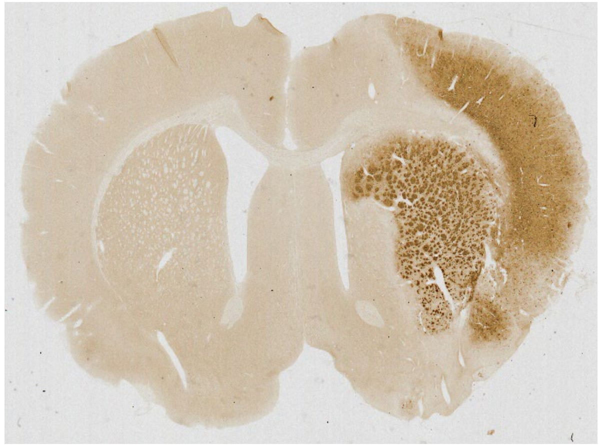

SDRM-422

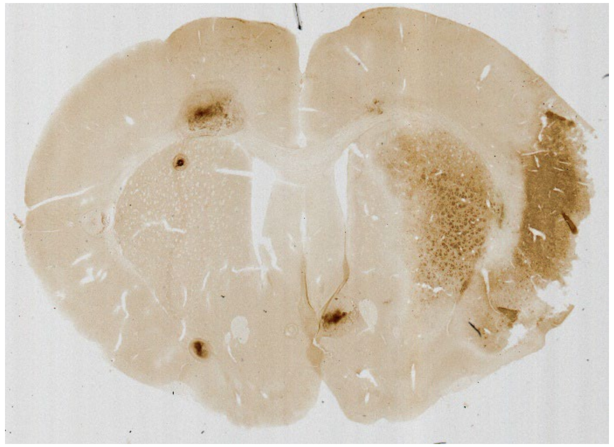

Grubb’s test identified outlier (non-specific staining in the contralateral hemisphere)

SDRM-436

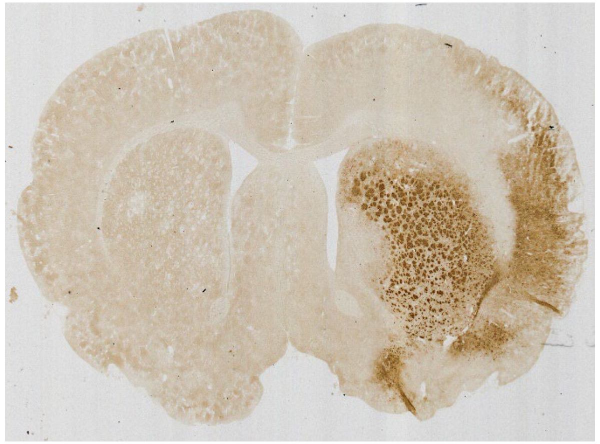

ImageJ Analysis of DAB-Stained Brain Sections

Figure 5B

Contralateral Hemisphere

Sham (3d)

Rat ID#	Total Area (mm2) of Contralateral Hemisphere	Stained Area (mm2) for B7-H3	Percentage of Stained Area in the Contralateral Hemisphere	Stained Area (mm2) for B7-H3	Mean Gray Scale Value	Inverse Mean Gray Scale Value	**Integrated Density**	IntDen (arbitrary value)
SDRM 369-0002.jpg	37.11	0.058	0.155	0.058	150.47	104.53	6.06	0.004
SDRM 375-0001.jpg	42.13	0.021	0.050	0.021	146.20	108.81	2.28	0.001
SDRM 376-0001.jpg	39.67	0.104	0.261	0.104	149.42	105.58	10.98	0.007
SDRM 384-0003.jpg	41.29	0.027	0.065	0.027	138.62	116.38	3.14	0.002
SDRM 394-0002.jpg	43.75	0.020	0.045	0.020	149.29	105.72	2.11	0.001
SDRM 467-0001.jpg	44.71	0.016	0.037	0.016	137.50	117.50	1.88	0.001

Stroke (2h-3d)

Rat ID#	Total Area (mm2) of Contralateral Hemisphere	Stained Area (mm2) for B7-H3	Percentage of Stained Area in the Contralateral Hemisphere	Stained Area (mm2) for B7-H3	Mean Gray Scale Value	Inverse Mean Gray Scale Value	**Integrated Density**	IntDen (arbitrary value)
SDRM 372-0029.jpg	36.091	0.061	0.168	0.168	149.72	105.29	17.69	0.011
SDRM 380-0030.jpg	37.641	0.036	0.095	0.095	144.97	110.03	10.45	0.006
SDRM 382-0032.jpg	38.391	0.033	0.086	0.086	143.82	111.18	9.56	0.006
SDRM 415-b(2)0044.jpg	41.151	0.026	0.062	0.062	138.61	116.39	7.22	0.004
SDRM 422-0041.jpg	33.714	0.551	1.634	1.634	157.78	97.22	158.86	0.096
SDRM 436-0042.jpg	40.131	0.066	0.165	0.165	153.27	101.74	16.79	0.010

Figure 5B

Ipsilateral Hemisphere

Sham (3d)

Rat ID#	Total Area (mm2) of Ipsilateral Hemisphere	Stained Area (mm2) for B7-H3	Percentage of Stained Area in the Ipsilateral Hemisphere	Stained Area (mm2) for B7-H3	Mean Gray Scale Value	Inverse Mean Gray Scale Value	**Integrated Density**	IntDen (arbitrary value)
SDRM 369-0002.jpg	39.268	0.07	0.19	0.073	151.63	103.37	7.55	0.005
SDRM 375-0001.jpg	40.259	0.03	0.08	0.033	147.55	107.45	3.55	0.002
SDRM 376-0001.jpg	39.829	0.07	0.18	0.071	153.77	101.23	7.19	0.004
SDRM 384-0003.jpg	39.812	0.03	0.09	0.034	145.54	109.46	3.72	0.002
SDRM 394-0002.jpg	45.704	0.07	0.15	0.071	148.35	106.66	7.57	0.005
SDRM 467-0001.jpg	45.079	0.02	0.05	0.022	143.70	111.30	2.45	0.001

Stroke (2h-3d)

Rat ID#	Total Area (mm2) of Ipsilateral Hemisphere	Stained Area (mm2) for B7-H3	Percentage of Stained Area in the Ipsilateral Hemisphere	Stained Area (mm2) for B7-H3	Mean Gray Scale Value	Inverse Mean Gray Scale Value	**Integrated Density**	IntDen (arbitrary value)
SDRM 372-0029.jpg	47.02	10.83	23.04	10.831	133.29	121.72	1318.30	0.799
SDRM 380-0030.jpg	47.64	6.94	14.58	6.943	132.65	122.35	849.50	0.515
SDRM 382-0032.jpg	44.93	9.68	21.56	9.684	128.21	126.79	1227.80	0.744
SDRM 415-b(2)0044.jpg	42.42	13.34	31.43	13.335	132.20	122.80	1637.56	0.992
SDRM 422-0041.jpg	36.04	7.64	21.21	7.644	135.64	119.36	912.38	0.553
SDRM 436-0042.jpg	43.41	11.68	26.91	11.680	132.33	122.67	1432.81	0.868
